# Cellulosome stoichiometry in *Clostridium cellulolyticum* is regulated by selective RNA processing and stabilization

**DOI:** 10.1038/ncomms7900

**Published:** 2015-04-24

**Authors:** Chenggang Xu, Ranran Huang, Lin Teng, Xiaoyan Jing, Jianqiang Hu, Guzhen Cui, Yilin Wang, Qiu Cui, Jian Xu

**Affiliations:** 1 Single-Cell Center, Qingdao Institute of Bioenergy and Bioprocess Technology, Chinese Academy of Sciences, Qingdao, Shandong 266101, China; 2 CAS Key Laboratory of Biofuels and Shandong Key Laboratory of Energy Genetics, Qingdao Institute of Bioenergy and Bioprocess Technology, Chinese Academy of Sciences, Qingdao, Shandong 266101, China; 3 University of Chinese Academy of Sciences, Beijing 100049, China

## Abstract

The mechanism, physiological relevance and evolutionary implication of selective RNA processing and stabilization (SRPS) remain elusive. Here we report the genome-wide maps of transcriptional start sites (TSs) and post-transcriptional processed sites (PSs) for *Clostridium cellulolyticum*. The PS-associated genes are preferably associated with subunits of heteromultimeric protein complexes, and the intergenic PSs (iPSs) are enriched in operons exhibiting highly skewed transcript-abundance landscape. Stem-loop structures associated with those iPSs located at 3′ termini of highly transcribed genes exhibit folding free energy negatively correlated with transcript-abundance ratio of flanking genes. In the cellulosome-encoding *cip-cel* operon, iPSs and stem-loops precisely regulate structure and abundance of the subunit-encoding transcripts processed from a primary polycistronic RNA, quantitatively specifying cellulosome stoichiometry. Moreover, cellulosome evolution is shaped by the number, position and biophysical nature of TSs, iPSs and stem-loops. Our findings unveil a genome-wide RNA-encoded strategy controlling *in vivo* stoichiometry of protein complexes.

## Abstract (web summary)

Selective RNA processing and stabilization (SRPS) can regulate bacterial operons, but the process is not well understood. Here, the authors show that the stoichiometry of cellulosome, a 12-subunit protein complex expressed from an operon in Gram-positive *Clostridium cellullolyticum*, is regulated by SRPS.

## Graphical abstract

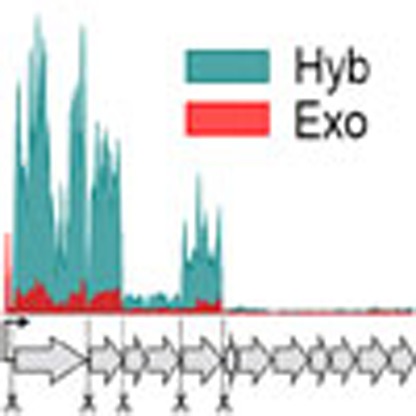

Many genes on genomes encode subunits of protein complexes, which carry out essential cellular activities or perform specialized functionality (for example, the cellulosomes that degrade cellulose^1^ or the photosystem complexes that harvest light^2^). In bacterial genomes, these genes are frequently organized as an operon, which is an efficient means to regulate transcription of multiple genes simultaneously. However, to create and maintain appropriate stoichiometry, functional complexes usually require accurate control of the relative abundance of subunit-encoding transcripts^3^. To resolve the potential conflict between the equimolar stoichiometry of transcripts within an operon and the non-equimolar stoichiometry of subunits frequently necessitated, one strategy employed by the cell is selective RNA processing and stabilization (SRPS)^4^, where the primary mRNA transcribed as an operon is processed by nucleases into segments first and then variation in stability among the segments contribute to differential gene expression^4^.

SRPS was reported for a number of Gram-negative bacterial operons that mostly encode protein complexes, such as *papA-I*^5^, malEFG^6^, CFA-I^7^, *unc*^8^, *ars*^9^, *ftsA-Z*^10^ and LEE4 (ref. 11) in *Escherichia coli*, and *rxcA*^12^ and *puf*^13^ in *Rhodobacter capsulata*. For most of the *E. coli* cases, the processing is initiated via a primary endonucleolytic cleavage by RNase E, a single-stranded, nonspecific endonuclease with preference for cleaving A/U-rich sequence^14^. Stability of the processed RNA can be influenced by factors such as the overall degree of translation, polyadenylation of mRNA 3′ termini, regulatory proteins and structural elements proximal to the 5′ or 3′ termini^15^. For example, 5′ stem-loops or 3′ intrinsic transcription terminators can significantly influence mRNA stability^14^,^16^. However, transcript-level heterogeneity among genes encoded within a single operon can also be caused by changes in operon structures or alternative transcriptional start sites (TSs; for example, in *Mycoplasma pneumonia*^17^ and *Anabaena*^18^). Therefore, it is not yet clear, at the genome-wide scale, how structure and function of the characteristic transcriptome of SRPS are dynamically regulated. Moreover, the global physiological roles of SRPS remain speculative, and whether the mechanism conveys specific competitiveness to the organism is not clear. Furthermore, on a macroscopic temporal scale, whether and how SRPS is implicated in organismal evolution are unknown. Finally, although Gram-positive and Gram-negative bacteria appear to employ distinct molecular machineries for SRPS^19^, experimental evidence for SRPS has been absent in Gram-positive bacteria (except for the *gapA* operon in *Bacillus subtilis*^20^).

*Clostridium cellulolyticum*, an anaerobic, mesophilic and cellulolytic Gram-positive bacterium, is one research model organism for microbial degradation of plant cell wall polysaccharides^21^,^22^,^23^. Like many other cellulolytic bacteria, *C. cellulolyticum* produces cellulosomes, which are high-molecular-mass protein complexes assembled from mostly extracellular enzymes^24^,^25^. *C. cellulolyticum* ATCC 35319 genome harbours a large number of glycosylhydrolase genes, and its ‘Cellulose Degradome' consists of a ‘core' set of 48 CAZymes required for degrading cellulose-containing substrates and an ‘accessory' set of 76 CAZymes required for specific non-cellulose substrates^26^. These genes are widely distributed along the 4.1-Mb genome and found in numerous operons^27^. Thus, efficient cellulolysis should rely upon a global regulatory network that controls the structure and abundance of such a large array of transcripts. However, the nature of this regulatory mechanism remains not fully understood^26^.

Here employing *C. cellulolyticum* H10 ATCC 35319 as a model, we present the first global landscape of SRPS regulation in a Gram-positive bacterial genome. Genome-wide RNA start sites that include both TSs and post-transcriptional processed sites (PSs) of transcripts were mapped at single-nucleotide resolutions via a differential mRNA-sequencing approach (dRNA-Seq). The 120 PS-associated genes are preferably associated with subunits of heteromultimeric protein complexes, and the 72 intergenic PSs (iPSs) are enriched in operons exhibiting highly skewed transcript-abundance landscape. Stem-loop structures associated with such iPSs are frequently found at the 3′ termini of highly transcribed genes, with their folding free energy negatively correlated with the transcript-level ratio between their flanking genes at a genome-wide manner. In the 12-gene *cip-cel* operon that encodes cellulosomal enzymes, *in vivo* and *in vitro* analyses of the primary and processed transcripts validated the predicted TSs and iPSs and unraveled a mechanism where a combination of five iPSs and four stem-loops precisely determine the stoichiometry of cellulosome *in vivo*. This mechanism is phylogenetically conserved and drives cellulosome evolution by specifying the structure and abundance of cellulosomal-subunit-encoding transcripts via varying the number, positioning and biophysical nature of TSs, iPSs and stem-loops. Our findings thus unveil a genome-wide, RNA-encoded mechanism linking sequences and *in vivo* stoichiometry of protein complexes, and have general implications in designing and engineering protein complex assembly.

## Results

### Genome-wide maps of TSs and PSs in *C. cellulolyticum*

**Figure 1:**
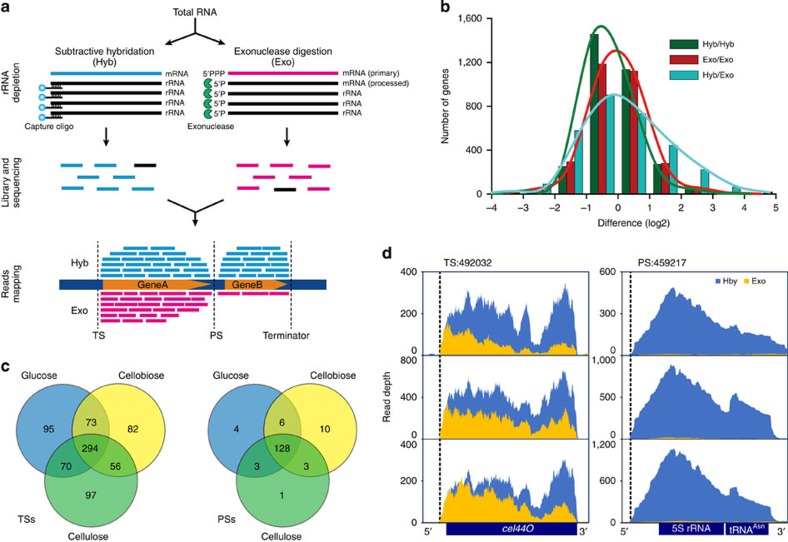
Genome-wide map of transcription start sites (TSs) and post-transcription start sites (PSs) in *Clostridium cellulolyticum*. (**a**) Two strategies were applied for purification of mRNA from total RNA (Methods). The primary and processed transcripts can be distinguished by comparison of results between the two methods. A transcript that has undergone processing would be characterized by a much lower level of transcript under Exo than under Hyb, whereas a transcript without undergoing processing would feature equivalent transcript level between the two libraries. Moreover, locations of the start of a primary transcript are indicated by an elevated and sharp 5′ flank under both Exo and Hyb, whereas the post-transcriptional processed-sites are indicated by an elevated and sharp 5′ flank under Hyb yet a much lower transcript level in the downstream flanking regions under Exo. (**b**) Histogram indicated, under cellulose, differences of transcript abundance between the two biological replicates of Hyb or Exo, or between Hyb and Exo. Compared with the symmetric distribution observed between biological replicates, the difference between Hyb and Exo exhibits dissymmetry distribution, suggesting certain genes are of lower transcript abundance in Exo than Hyb (due to the sensitivity of processed transcripts to exonuclease). (**c**) TSs and PSs identified under the culture conditions of cellulose, cellobiose or glucose. Their overlaps were showed via Venn diagram. (**d**) Transcription profiles of the primary transcript of *cel44O* and the processed transcript of 5S rRNA-tRNA^Asn^. Exonuclease treatment (Exo) shifts the *cel44O* cDNAs towards the nuclease-protected 5′ end, yielding a sawtooth-like profile with an elevated sharp 5′ flank. In contrast, the processed 5′ end of 5S rRNA is predominant in the Hyb.

Mapping of TSs and PSs requires the ability to discriminate between primary and processed transcripts. In dRNA-Seq, a pair of differential cDNA libraries was sequenced, with the Hyb library selecting for all primary and secondary mRNA, whereas the Exo library selecting for only primary transcripts (Methods). Primary and processed transcripts are then distinguished by comparing results from the two libraries (Fig. 1a and Supplementary Methods).

To maximize the coverage of transcripts sampled, *C. cellulolyticum* was cultivated under each of the three carbon sources of cellulose, cellobiose and glucose. The extracted total RNA was, respectively, treated by Hyb and Exo and then sequenced. A total of 73.2 million reads were uniquely mapped to the genome, representing combined coverage of 791X. After removing rRNA reads, from 73.5 to 87.8% of the reads were mapped to coding sequence (CDS), and the remaining were either upstream of CDS (thus putatively identifying a 5′-untranslated region (5′-UTR)) or mapped to unannotated or potentially mis-annotated regions (Supplementary Table 1).

The transcript abundance (TA) of each gene under a particular condition was determined based on trimmed mean of *M* values (Supplementary Data 1). For each of the two treatments (Hyb and Exo) under each of the three carbon sources, correlation of the global TA among biological replicates was high (*r*^2^ from 0.872 to 0.958; Supplementary Fig. 1), yet that between the Hyb and Exo treatments was much lower (*r*^2^ from 0.771 to 0.869; Supplementary Fig. 1). This is due to the lower abundance of certain transcripts in Exo: for example, under cellulose, for 24% of genes the abundance under Exo is <50% of that under Hyb, while merely 12 or 10% of genes were found that way between the biological replicates under Exo or Hyb (Fig. 1b). A certain portion of those genes exhibiting lower TA in Exo than Hyb should represent processed transcripts, as they were specifically digested in the Exo library by the 5′-monophosphate-dependant exonuclease that recognizes their terminal 5′-monophosphate.

To identify TSs and PSs genome-wide, 1408 RNA end-sites (ESs) were first annotated based on ES localization positions (Supplementary Methods). Furthermore, by comparing the numbers of reads mapped to ES between Hyb and Exo libraries, 767 TSs (Supplementary Data 2) and 155 PSs (Supplementary Data 3) were found. Among them, 517 TSs plus 135 PSs (under cellulose), 505 TSs plus 147 PSs (under cellobiose) and 532 TSs plus 141 PSs (under glucose) were identified, respectively, with 294 TS and 128 PS shared among all three conditions (Fig. 1c). The TSs were grouped into three categories based on genomic context (Supplementary Fig. 2): (i) 663 gTSs (that is, ‘gene' TSs), which are located upstream of a gene; (ii) 63 iTSs (that is, ‘internal' TSs), which are positioned within a CDS; (iii) 41 nTSs (that is, ‘non-coding' TSs), which are orphans with no annotated genes in proximity.

The identified primary transcriptional starting and post-transcriptional processing events were supported by the several known ones such as TS:492032 and PS:459217 (Supplementary Data 2 and 3), which, respectively, start the primary transcript of cellulosomal cellulase *cel44O* (ref. 28) and the processed transcript of 5S rRNA^19^. Transcriptional profiles of *cel44O* that harbour TS under all three substrates are towards a sawtooth-like profile and with an elevated and sharp 5′ flank in Exo and Hyb, yet the processed transcripts of 5S rRNA-tRNA^Asn^ were nearly completely digested in Exo (Fig. 1d).

### Operons with complex transcript-abundance ratio of genes

**Figure 2:**
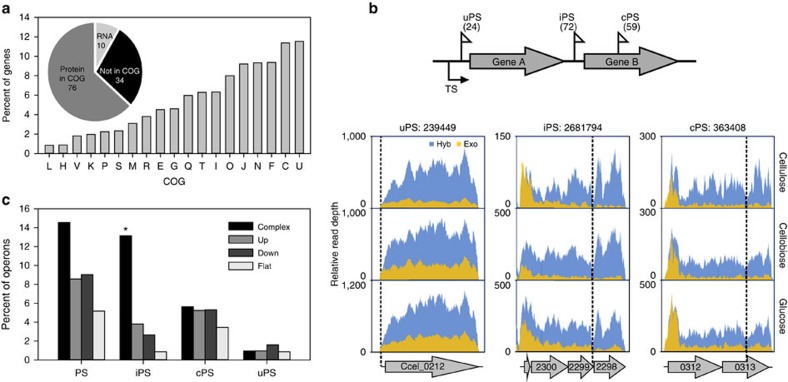
Functional analysis of genes that are associated with post-transcription start sites (PSs). (**a**) Functional profile of these genes, which include 10 RNA genes and 110 protein-coding genes. Among the 110 protein-coding genes with COG assignments^29^, 48 genes encode subunits of known protein complexes. Percentages of such genes in each COG term were shown in columns. Genes are assigned to COG terms based on function. (**b**) Top: classification of PSs based on their location in the operon: 5′-UTR (uPS), intergenic spacers (iPS) or CDS (cPS). Bottom: one example of each of uPS, iPS and cPS was shown. (**c**) Distribution of operons that harbour PSs, iPSs, cPSs or uPSs among the Down, Flat, Up and Complex types of operons. The iPSs were enriched in the Complex type of operons (*P value <0.0001, hypergeometric test).

To probe the functional implication of PSs, the 110 protein-coding genes that are associated with PSs were examined for association with functional categories as defined by Cluster of Orthologous Groups^29^ (COG; Supplementary Data 3). A broad spectrum of COGs was found (34 of the 110 were not assigned to any COGs), with these genes being enriched in COG categories such as U, C, F, N and J (Fig. 2a). Among the 120 PS-associated genes, 110 encode proteins, among which 85 encode proteins with annotated function. Over half of such genes (48 of 85) encode subunits of known protein complexes.

Protein complexes are frequently encoded as operons in bacterial genomes, thus it is possible that PSs are linked to operon-based gene regulation. To test this hypothesis, the relative positions of PSs in operons genome-wide were determined. An operon map was produced by integrating the primary transcriptional starts from our experimentally determined TS map with the *in silico* predicted *C. cellulolyticum* operons in DOOR (Database of prOkaryotic OpeRons^30^), followed by testing the contiguity of primary transcripts across all intergenic regions using our RNA-Seq data. This first experimentally determined operon map in cellulolytic *Clostridium* spp. consists of 1,810 operons (harbouring 3,569 genes; Supplementary Data 4), among which 1,062 (58.7%) are monocistronic and 748 (41.3%) are polycistronic, with 380, 157, 90 and 121 operons harbouring two, three, four and over four genes, respectively. Overlaying the operon structures onto the gene-specific transcript level landscape revealed differential TA among the genes within operons. For example, 188 and 116 of the 748 poly-cistronic operons, respectively, exhibited the ‘Down' and the ‘Flat' patterns that are consistent with natural polarity^17^, and 210 showed reversed polarity and exhibited the ‘Up' pattern, whereas 213 exhibited ‘Complex' patterns that feature non-linear relationship between the order of a gene and its transcript level (Supplementary Data 4).

Via this operon map, the 155 PSs were mapped into 93 operons (among which 75 are poly-cistronic operons; Supplementary Data 4). Based on their physical positions relative to the coding regions in an operon, the PSs can be classified into three categories (Fig. 2b and Supplementary Data 3): (i) cPSs (‘coding PSs'; 59 in total), which are positioned internally within annotated CDS; (ii) uPSs (‘UTR PSs'; 24), which are located at the 5′-UTR of operons; (iii) iPSs (‘intergenic PSs'; 72), which are located at intergenic regions and can potentially guide cleavages that physically uncouple genes encoded on a polycistronic mRNA. Interestingly, operons with ‘Complex' patterns (13.2% of them) are much more likely to harbour iPSs than those with the other patterns (2.7% of them in Down, 0.9% in Flat and 3.8% in Up). Thus, the iPSs (but not uPSs or cPSs) are specifically associated with complex operons (*P* value<0.0001; hypergeometric test; Fig. 2c).

**Figure 3:**
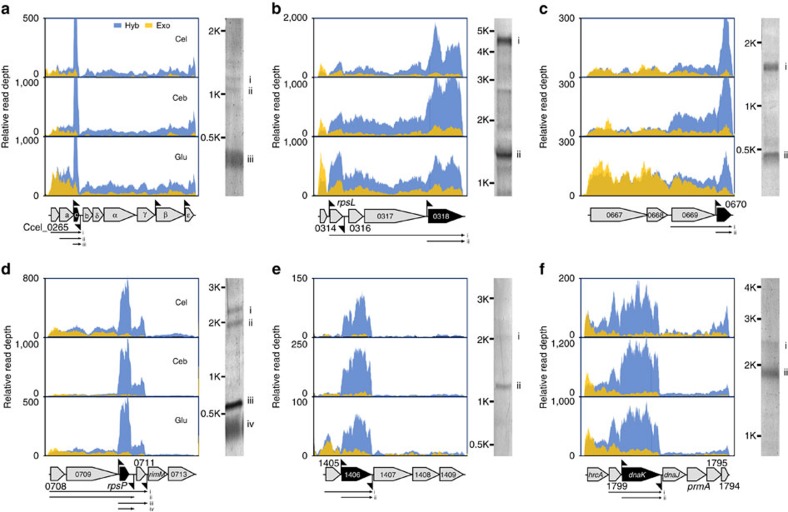
Transcription profiles of the iPS-harbouring operons of the complex type. (**a**) Ccel_0265-0273, which encodes subunits of F-type ATPase. (**b**) Ccel_0314-0318, which encodes subunits of ribosome. (**c**) Ccel_0667-0670, which encodes subunits of indolepyruvate oxidoreductase. (**d**) Ccel_0708-0713, which encodes proteins related to ribosome function. (**e**) Ccel_1406-1409, which encodes subunits related to tansporter function. (**f**) Ccel_1800-1794, which encodes proteins related to molecular chaperone function. The northern blotting results were shown next to the profiles. Location and direction of the iPSs were indicated by pennants. Black block arrows specified those genes that were selected as templates for designing northern blotting probes. Roman numerals beneath the operon indicated the transcripts with the expected length as derived from dRNA-Seq, whose corresponding bands were highlighted on the gel images of northern blotting.

Intriguingly, 69.4% of the iPSs are located either upstream or downstream of the 37 genes that exhibit the highest transcript level among all genes in their respective operons. Over two-third (26) of these 37 genes are located in the middle or 3′ terminal of their respective operons (six examples shown in Fig. 3 and Supplementary Data 3). Six of such genes were selected as template for preparation of Digoxigenin (DIG)-labelled probes for northern blot, which validated the existence of iPSs in the transcripts of these operons (Fig. 3a–f). In each of the operon, a monocistronic transcript of the target gene in its corresponding size was respectively detected; in addition, other transcripts with sizes consistent with the location of PS were detected, supporting cleavage of the primary transcript into various transcripts harbouring the target gene at the PSs. Furthermore, the highest transcript level of the target gene was confirmed for each of the operon. For example, the Ccel_0265-0273 operon that encodes F-type ATPase is transcribed as a polycistronic RNA, but the third gene that encodes the *c* subunit of ATPase and harbours two iPS in its 5′ and 3′ terminals is of the highest transcript level (three- to five-folds of its flanking genes; Fig. 3a). The observed ratio of transcript level is likely biologically significant as it is consistent with reported stoichiometry of the protein complex where the relative dose of the eight subunits (alpha, beta, gamma, delta, epsilon, *a*, *b* and *c*) is 3:3:1:1:1:1:2:10 (ref. 31). Therefore, the iPSs are potentially implicated in the observed complex abundance ratio of processed transcripts within an operon.

### Role of iPSs and their associated stem-loops

**Figure 4:**
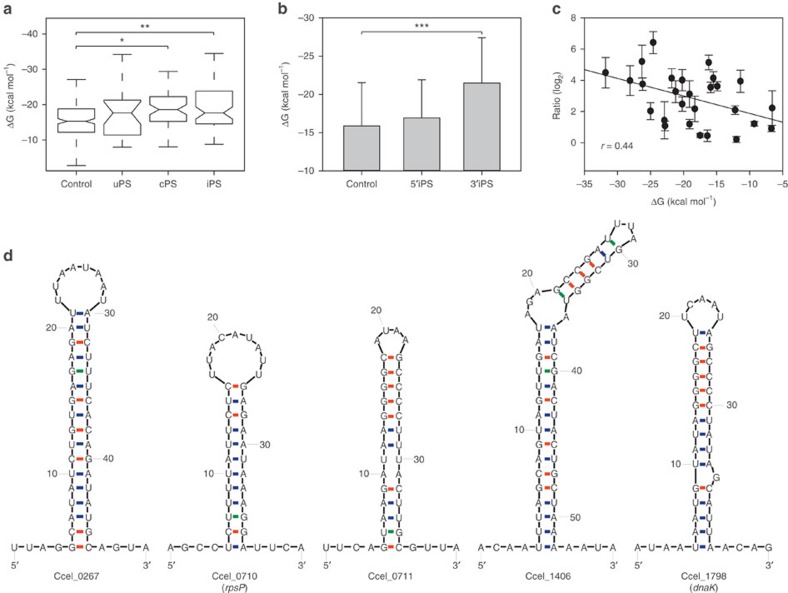
Mechanism underlying the distinct ratio in transcript abundance among the genes in ‘Complex' type operons. (**a**) The RNA folding free energy (Δ*G*) of sequences flanking uPSs (*N*=24), cPSs (*N*=59) and iPSs (*N*=72). Sequences from non-PS intergenic regions within operons were used as the control (*N*=195). *P value=0.0005; **P value=0.0001 (Student's *t*-test). (**b**) Comparison of the average Δ*G* between the 5′iPS-proximal sequences (*N*=41) and the 3′iPS-proximal sequences (*N*=31). Sequences from non-PS intergenic region within operons were as the control. Error bars indicate s.d. of mean of all sequences in each type (***P value<0.0001, Student's *t*-test). (**c**) Correlation of the degree of transcript protection with folding free energy (Δ*G*) of the 3′iPS associated stem-loops. The degree of protection was quantified by the ratio in transcript abundance between the upstream and downstream genes that flank the iPSs. (**d**) The 3′iPS associated stem-loop structures that are, respectively, located at the 3′ end of Ccel_0267, *rpsP*, Ccel_0711, Ccel_1406 and *dnaK*. These structures are found in the operons shown in Fig. 3.

To probe the role of iPSs in processing of polycistronic mRNA, RNA secondary structure of PS-proximal sequences was compared with that of random intergenic sequences using Mfold^32^ (Supplementary Data 3 and 5). The average folding free energy of sequences (Δ*G*) around PSs, at −18.75 kcal mol^−1^, is significantly lower than the control (−15.88 kcal mol^−1^). Moreover, sequences around iPSs exhibit the lowest average Δ*G* (−19.32 kcal mol^−1^; *P* value=0.0001; Student's *t*-test; Fig. 4a). However, only the average Δ*G* of 3′iPS-proximal sequences (−23.45 kcal mol^−1^), but not that from 5′iPSs (−16.19 kcal mol^−1^), is significantly lower than the control (*P* value<0.0001; Student's *t*-test; Fig. 4b).

For 45.8% of the iPSs, a putative stem-loop structure (33 in total) was found within 100 bp from the iPS, with their Δ*G* ranging from −6.6 to −31.8 kcal mol^−1^ (Fig. 4c and Supplementary Data 3). Among the predicted stem-loops, 27 are associated with 3′ iPSs, whereas only 6 are associated with 5′ iPSs. This could explain the significantly lower average Δ*G* from 3′PS-proximal sequences than the 5′PS-proximal sequences.

Sequence analysis suggested that majority of the 33 iPSs stem-loops are likely not the classical rho-independent transcriptional terminators. First, only 5 of the 33 are followed by a run of U residues, which is a distinct feature of the rho-independent terminator^33^; the majority (that is, 28 of the 33) do not carry the feature. Second, 7 of the 33 were predicted as potential rho-independent terminators by TransTermHP^34^ (including the two followed by a run of U residues); however, the genes downstream of each of these seven stem-loops in the same operon were still transcribed, which argues against their role as rho-independent terminators.

Intriguingly, the presence of such 3′PS-proximal stem-loops is strongly associated with the higher transcript level of their associated genes. For example, five genes that harbour 3′PS stem-loops (Ccel_0267, *rpsP*, Ccel_0711, Ccel_1406 and *dnaK*; Fig. 4d) each exhibited the highest transcript level among all genes in their respective operons (Fig. 3). Moreover, the transcript-abundance ratios between processed transcripts harbouring 3′iPS stem-loops and their 3′iPS flanking transcripts were negatively correlated with Δ*G* of the stem-loops involved (*r*=0.44; Fig. 4c). Altogether, these observations suggested that main protection for the processed transcripts is due to their 3′ end, in that higher stability of stem-loops as determined by the 3′end sequence would promote higher abundance of the associated processed transcripts. Thus, these stem-loops are potentially the mechanistic link between iPSs and the observed complex ratio of processed transcripts derived from an operon.

### Regulation of cellulosome stoichiometry

**Figure 5:**
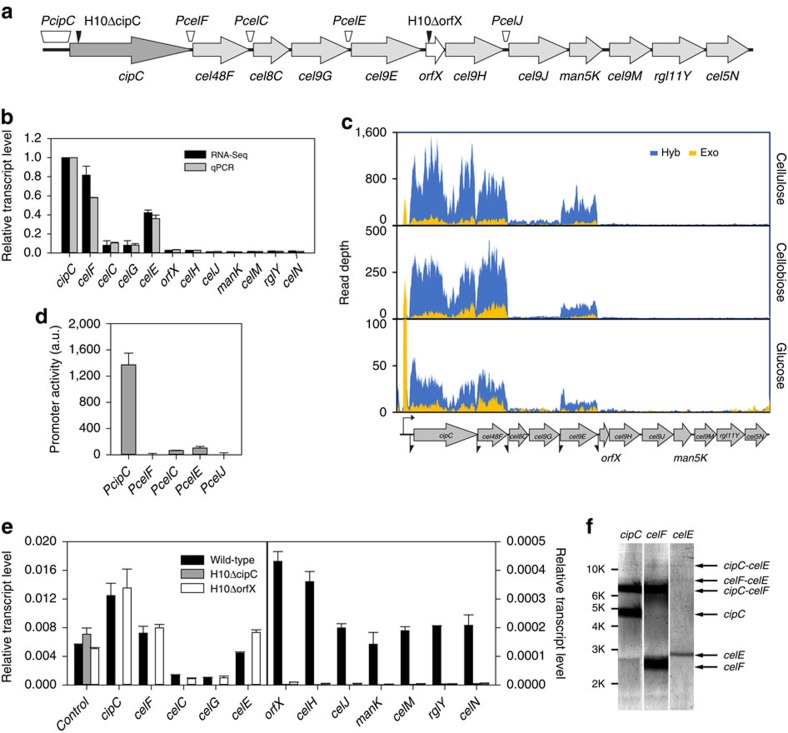
The *cip-cel* operon is regulated by the SRPS mechanism. (**a**) Organization of the *cip-cel* operon. Those regions that underwent analysis of promoter activity and those sites where insertion mutagenesis took place are indicated. (**b**) The ratio in transcript level among the 12 genes in the *cip-cel* cluster. The ratio was first measured by RNA-Seq and then validated by qPCR. (**c**) Comparison of transcriptional landscapes of the *cip-cel* operon from Hyb library and Exo library. TSs are indicated by bent arrows and iPSs by scissors. (**d**) Analysis of the promoter activity of intergenic regions of the *cip-cel* cluster using the FbFP reporter. A.U.: artificial unit. (**e**) Transcription of *cip-cel* cellulosomal genes (Ccel_0728-0740) under cellobiose in the *cipC* and *orfX* knockout mutants. The transcript levels of H10ΔcipC and H10ΔorfX and the wild-type were measured via qPCR and compared. Ccel_0750 was selected as control. In the insertion mutagenesis mutants, no transcripts derived from those genes downstream of the mutated sites in the *cip-cel* cluster were detected. (**f**) Northern blotting analysis of transcripts carrying the genes of the *cip-cel* operon. Error bars indicate s.d. of mean of experiments in triplicate.

The *cip-cel* cluster was employed as a model to probe the physiological role of such mechanisms underlying the differential transcript level intraoperon. It harbours 12 genes that encode the major cellulosomal subunits^35^: the scaffoldin (Ccel_0728), 4 CAZymes (Ccel_0729-0732), an unknown protein (Ccel_0733) and then another 6 CAZymes (Ccel_0734-0740; Fig. 5a). The mRNA-Seq data revealed that under all the three carbon sources tested these 12 genes exhibit highly skewed TA (a ratio of 100:110.2:8.6:8.1:38.1:5.0:3.9:2.0:2.6:2.2:3.2:4.7; Fig. 5b), where the observed transcript level of the first (Ccel_0728, encoding the scaffoldin CipC), second (the Cel48F-encoding Ccel_0729, an exoglucanase of GH48) and fifth (the Cel9E-encoding Ccel_0732, an endoglucanase of GH9) genes were far higher than (>5 times of) the other genes in the cluster (these results were validated by quantitative PCR (qPCR); Fig. 5b). Interestingly, this ratio is independent of the carbon sources, as demonstrated by the high correlation among the ratios measured under cellulose, cellobiose and glucose (*r*^2^ ranged from 0.868–0.989; Supplementary Table 2). Such a highly skewed transcript ratio in the *cip-cel* cluster has functional implications, as it was correlated with the relative abundance of the encoded proteins (*r*^2^=0.560; Supplementary Fig. 3) and was consistent with observation that CipC, Cel48F and Cel9E are the most abundant proteins in purified *C. cellulolyticum* cellulosome^23^,^26^,^36^. Therefore, a mechanism that regulates the stoichiometry of cellulosome should be present.

Our mRNA-Seq data revealed that all genes in the *cip-cel* cluster were transcriptionally linked, suggesting formulation of the cluster as a single operon (Fig. 5c). The TS map revealed that the cluster only harboured one TS located 643 bases upstream from the ATG codon of the first gene (*cipC*) and included no TSs in the other intergenic regions of the cluster (Fig. 5c and Supplementary Data 2). To verify this, promoter activity of all intergenic regions of the operon was evaluated using a fluorescent reporter system, where the 5′ intergenic regions of *cipC*, *cel48F*, *cel8C*, *cel9E* and *cel9J* were, respectively, fused to the reporter gene *fbfp* and separately transformed into *C. cellulolyticum* for measurement of fluorescence during mid-log phase of growth on cellobiose. None of the intergenic regions exhibit promoter activity except for the 5′ intergenic region of *cipC* (Fig. 5d), confirming that the *cip-cel* operon was transcribed by the single promoter located upstream of the 5′ end of *cipC*.

To rule out the presence of within-CDS promoters, two genes (*cipC* and *orfX*), which are, respectively, the first gene of the highly transcribed front half (*cipC*-*cel9E*) and the poorly transcribed rear half (*orfX*-*Cel5N*) of the *cip-cel* cluster, were each disrupted by intron. In the two mutants H10ΔcipC and H10ΔorfX, the genes downstream of mutations in the cluster were not transcribed, but the control gene Ccel_0750 was (Fig. 5e). Moreover, none of the transcripts of *cip-cel* genes was detected in the H10ΔcipC strain, consistent with previous observations that a mutant with insertion mutation of *cipC* failed to express any of the *cip-cel* enzymes^37^,^38^. Thus, the 12-gene cluster is a single transcriptional unit. Together, these results ruled out contribution of any TSs to the highly skewed transcript landscape in the *cip-cel* cluster.

On the other hand, dRNA-Seq suggested that the primary *cip-cel* transcript harbours at least five PSs: one uPS (upstream of *cipC*) and four iPSs (between *cipC* and *cel48F*, *cel48F* and *cel8C*, *cel9G* and *cel9E*, *cel9E* and *orfX*, respectively; Fig. 5c and Supplementary Data 3). Northern blotting with probes, respectively, targeting *cipC*, *cel48F* and *cel9E* revealed that (Fig. 5f), besides the monocistronic transcripts of *cipC* (4.9 kb), *cel48F* (2.3 kb) and *cel9E* (2.8 kb), polycistronic transcripts of approximately 7.6, 8.7 and 14 kb were detected, agreeing with the expected sizes of the *cipC-cel48F*, *cel48F-cel9E* and *cipC-cel9E* portions of the operon (Fig. 5f). Interestingly, one stem-loop was located downstream of each of *cipC*, *cel48F*, *cel9G* and *cel9E* (Supplementary Fig. 4a), which might underlie their much higher TA than their flanking genes (which are free of stem-loops).

**Figure 6:**
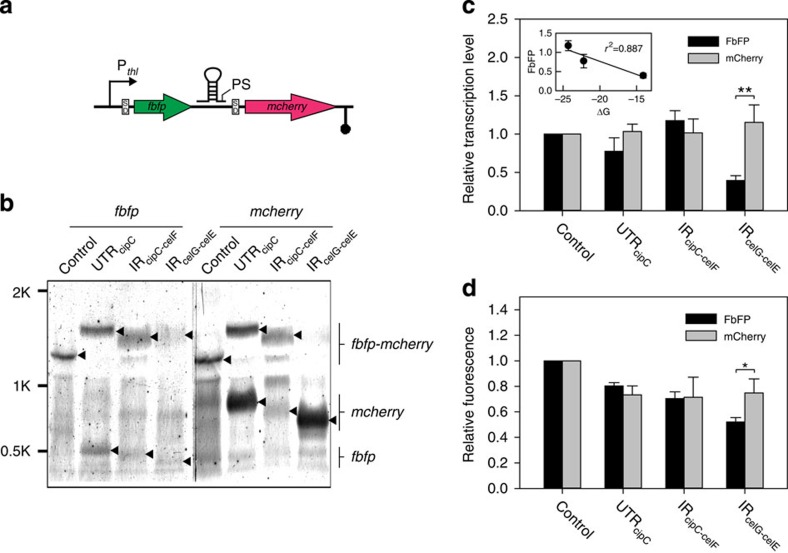
Functional analysis of iPS-proximal stem-loops located in the *cip-cel* operon via *fbfp*-*mcherry* artificial operon. (**a**) Schematic representation of the dual-fluorescence reporter system for functional analysis of stem-loop structures. (**b**) Northern blotting results that yield the size and abundance of transcripts that harboured *fbfp*, *mcherry* or both. Black triangle arrows highlight positions of bands that corresponded to the transcripts as indicated on the right side of the panel. (**c**) The relative transcription level of *fbfp* and *mcherry* as measured by qPCR. Data were normalized via the transcript level of the corresponding gene in the control, which does not harbour any iPS proximal sequences. The inset shows the strong correlation between the transcript level of FbFP and Δ*G* of the inserted stem-loops (*r*^2^=0.887). (**d**) The fluorescence intensity of FbFP and mCherry proteins as normalized by OD_600_. Data were then normalized via the fluorescence in the control. Error bars indicate s.d. of mean of experiments in triplicate (*P value<0.01, **P value<0.0001; Student's *t*-test).

To probe this hypothesis, an experiment testing *in vivo* role of the stem-loops was designed, where three 92- to 231-bp-long genomic segments each located in one of the three regions below were amplified: (i) 5′-UTR region of *cipC* (UTR_cipC_, including a secondary structure with ΔG of −22.2 kcal mol^−1^ and uPS 838189; 231 bp), (ii) intergenic region between *cipC* and *cel48F* (IR_cipC-celF_, including a stem-loop with ΔG of −24.3 kcal mol^−1^ and iPS 842939; 116 bp) and (iii) intergenic region between *cel9G* and *cel9E* (IR_celG-celE_, including a stem-loop with Δ*G* of −14.1 kcal mol^−1^ and iPS 849042; 92 bp). Each of the three segments was then, respectively, inserted between the reporter genes *fbfp* (encoding a green fluorescence protein) and *mcherry* (encoding a red fluorescence protein; Fig. 6a). The resulted artificial operons (or the control, where no segments were inserted) were then separately introduced into *C. cellulolyticum*.

In northern blotting, two probes were designed to detect transcripts that, respectively, harbour *fbfp* and *mcherry*. Only a single 1.2-kb band from the control (which harbours no PSs) was detected by both probes (Fig. 6b), consistent with the expected size of bicistronic transcript of *fbfp-mcherry*. In addition to the full-size band of the biocistronic transcript, monocistronic transcripts of *fbfp* and *mcherry* with their predicted sizes were also detected in each of the three artificial operons that carry PS sequences (Fig. 6b), suggesting occurrence and positions of the RNA processing as predicted. Thus, the predicted PSs in each of the three segments were functional.

Moreover, qPCR revealed that the relative TA of *fbfp* was 66% lower than that of *mcherry* for IR_celG-celE_ (*P* value<0.0001; Student's *t*-test), but not for UTR_cipC_ and IR_cipC-celF_ (Fig. 6c). This was consistent with the observation in the *cip-cel* operon *in vivo* (Fig. 5c). Thus, the secondary structures promoted the abundance of processed transcripts, presumably by protecting the transcripts from RNase digestion.

Furthermore, as quantified via the fluorescence signals, protein abundance of *fbfp* was significantly lower than that of *mcherry* for IR_celG-celE_ (*P* value<0.01; Student's *t*-test), but not for UTR_cipC_ and IR_cipC-celF_ (Fig. 6d). Thus, for the regulated genes, relative abundance of proteins was consistent with that of transcripts, suggesting biological implication of the post-transcriptional regulation.

Interestingly, at both transcript and protein level, two observations were apparent. First, the TAs of *fbfp* which harbours a free 3′ end are distinct among the three strains, however, that of *mcherry*, which harbours a free 5′ end remained unchanged (relative to that in the control), suggesting that the introduced sequences impact transcript level of their upstream gene but not their downstream gene. Second, level of the *fbfp* transcripts was correlated with Δ*G* of stem-loops (*r*^2^=0.887), with the relative TA of *fbfp* increasing from 0.39 to 1.18, whereas the Δ*G* decreasing from −14.1 to −24.3 (Fig. 6c). Together, the evidence suggested that the stem-loops can control abundance of the processed transcript of its upstream gene, that is, *cipC* and *cel9G*.

**Figure 7:**
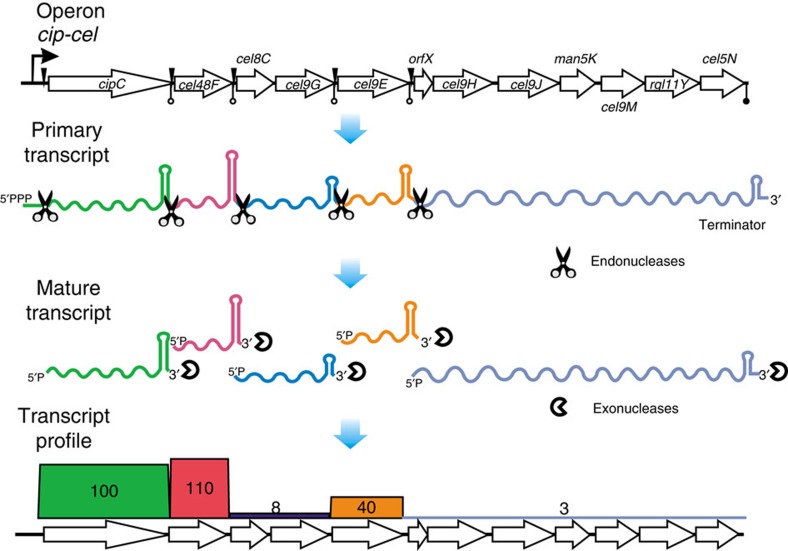
A model for regulation of the stoichiometry of cellulosomal components *in vivo*. The *cip-cel* operon is transcribed by its sole promoter and the primary transcript is cleaved into several secondary transcripts by endonucleases as defined by iPSs. However, stability of these secondary transcripts against exonuclease degradation varied due to their distinct terminal structure. The resulted distinction in transcript level among the genes result in the observation of a ‘complex' type operon and eventually lead to the proper composition and ratio of cellulosome subunits in *C. cellulolyticum*.

These findings, together with inter-operon regulation that we previously revealed for the 148 CAZymes in *C. cellulolyticum*^26^, allowed us to propose a post-transcriptional regulatory model for cellulosomal loci (Fig. 7). Under the control of Carbon Catabolite Repression, the *cip-cel* operon is transcribed by its sole promoter into the primary *cip-cel* mRNA^26^. Cleavage signals located in the intergenic regions are specifically recognized by endonucleases (such as RNase E or RNase Y)^39^, resulting in cleavage of the primary transcript into at least six secondary transcripts. Stability of these secondary transcripts varied widely due to their distinct terminal sequences that convey resistance to exonuclease degradation (such as PNRase, RNase II or RNase R), with the Δ*G* of stem-loop structure located at the ends of processed transcripts (such as *cipC*, *cel48F*, *cel9G* and *cel9E*) quantitatively determining the observed TA. This model explains the highly skewed ratio of TA among the *cip-cel* genes, which eventually results in the proper protein recipe (that is, profile and ratio of subunits) of cellulosomes.

### A mechanism for cellulosome evolution in *Clostridium* spp

**Figure 8:**
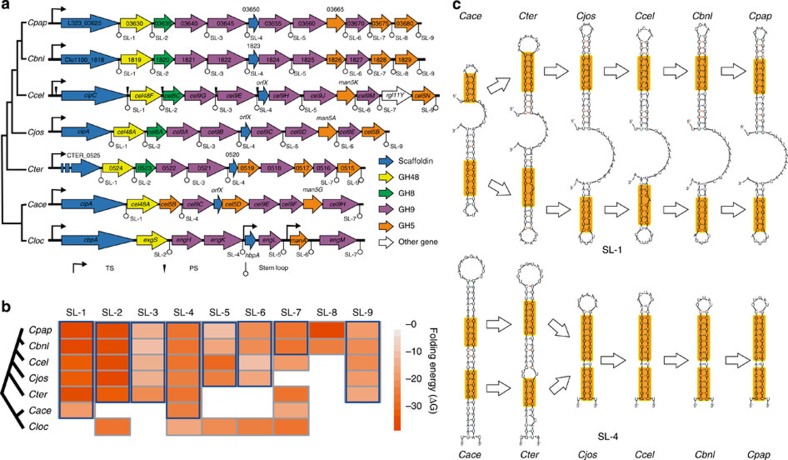
Mechanism driving the evolution of cellulosomal loci in the seven *Clostridium* spp. (**a**) Organization of the orthologous *cip-cel* loci in seven mesophilic *Clostridium* spp. that include *C. cellulovorans* (*Cloc*), *C. acetobutylicum* (*Cace*), *C .papyrosolvens* (*Cpap*), *C. josui* (*Cjos*), *C. sp*. BNL1100 (*Cbnl*), *C. termitidis* (*Cter*) and *C. cellulolyticum* (*Ccel*). Gene name, encoded function and locations of TS, PS and stem-loop structure were shown. Phylogenetic tree of the seven organisms (based on 16S sequences) was shown on the left. (**b**) Energy comparison and evolution of the stem-loop structures located in the cellulosomal loci from the seven *Clostridium* spp. Folding free energy (Δ*G*) of stem-loop structures was indicated via the depth of colour in the heat map, in which depth of coloured boxes represents the folding free energy of stem-loops. Orthologous stem-loops are marked in blue boxes. (**c**) Evolution of sequence and structure of SL-1 orthologues and SL-4 orthologues from the six *Clostridium* spp. (other than *Cloc*). Their conserved sequences were shown in orange boxes.

*Cip-cel*-like clusters were also present in another six mesophilic *Clostridium* spp.: *C. papyrosolvens*, *C. sp*. BNL1100, *C. josui*, *C. termitidis*, *C. acetobutylicum* and *C. cellulovorans* (Fig. 8a). These clusters are orthologous^35^, because of similarity in structure, functional correlation of encoded proteins, orthology among most of the genes and correlation between operon organization and organismal phylogeny (Fig. 8a).

Intriguingly, orthologous stem-loops were found in the intergenic regions of these cellulosomal clusters (Fig. 8b and Supplementary Data 6). First, comparison of the profile and organization of stem-loops suggested the presence of at least eight orthologous stem-loops among the six *Clostridium* spp. (other than *C. cellulovorans*). Thus, the six clusters that harbour these orthologous stem-loops are likely controlled by SRPS, but the *C. cellulovorans* cluster. Consistent with this, genes in the *C. cellulovorans* cellulosomal cluster were found transcriptionally controlled by multiple internal promoters (for example, for the first, fifth and seventh genes, respectively)^40^.

Second, *C. acetobutylicum* features a cellulosomal cluster whose structure and sequence of the stem-loops are more similar to *C. cellulolyticum* than to *C. cellulovorans* (despite its closer phylogenetic relationship with the later; Fig. 8b,c), suggesting that the *C. acetobutylicum* cluster is likely regulated by the SRPS mechanism.. In addition to the terminator located at its 3′ terminal, the *C. acetobutylicum* cluster harbours merely two stem-loops (the lowest number among the six clusters), which are SL-1 and SL-4 orthologues and are located at 3′ terminal of the first gene (*cipA*) and the third gene (*cel5B*), respectively (Fig. 8b). These two *C. acetobutylicum* stem-loops are of the highest Δ*G* and thus the least stable among their orthologues (Fig. 8c). Moreover, in each of the other *C. cellulolyticum*-type clusters, two additional stem-loops were found in 3′ terminal of the GH48 and GH9 family genes (for example, *cel48F* and *cel9E* in *C. cellulolyticum*, which as representatives of GH48 and GH9 family cellulases are the most important in hydrolysis of crystalline cellulose^41^). Consequentially, the transcripts of *cel48F* and *cel9E* are protected, resulting in the highest transcript and protein level among the *cip-cel* genes in *C. cellulolyticum*. Thus, the *C. acetobutylicum* cluster appears to represent a primitive or dysfunctional stage of SRPS, which lacks the two key stem-loops for protection of *cel48F* and *cel9E* transcripts. This may potentially explain why this bacterium reknown for the Acetone Butanol Ethanol fermentation process is not cellulolytic, despite possession and expression of the cellulosomal cluster^42^.

Third, the stem-loops of cellulosomal loci have also been dynamic during evolution. With the intergenic sequence grew, new stem-loop structures emerged and eventually created the characteristic operon configuration that underlies SRPS. For example, the number of stem-loop grew from two (in *C. acetobutylicum*) to eight (in *C. papyrosolvens* and *C. sp*. BNL1100; Fig. 8b), whereas structure of the stem-loops became increasingly compact (that is, with fewer bulges and smaller loops; Fig. 8c), with several (for example, SL-1 and SL-6) undergoing gradual elevation in Δ*G* while others (for example., SL-3 or SL-5) experiencing reduction in Δ*G* (Fig. 8b). Therefore, SRPSs, guided by gain/loss of stem-loops and elevation/reduction of their stability, have driven the evolution of cellulosomes in *Clostridium* spp.

## Discussion

Probing the mechanisms responsible for RNA processing has been challenging, mainly due to the rapid decay of most bacterial mRNAs^43^. Although several studies measuring mRNA half-lives using microarray were reported^44^,^45^,^46^, genome-wide mapping of RNA-processed sites for bacteria have not been well established. Thus, this first genome-wide TS and PS map in Gram-positive bacteria provides a framework for analysing RNA processing or delay both genome-wide and at individual loci.

Our genome-wide profiling of SPRS in the Gram-positive *C. cellulolyticum* revealed certain global features shared with that in Gram-negative bacteria. First, the PSs in *C. cellulolyticum* were assigned into over 120 gene transcripts from a wide spectrum of functional categories, similar to the global impact of endoribonuclease RNase E in *E. coli* and RNase Y in *B. subtilis* on mRNA turnover. Second, SRPS-controlling operons in *C. cellulolyticum* mostly encode protein complexes, which is consistent with the individually reported cases in Gram-negative bacteria^5^,^6^,^7^,^11^. Orthologues of several SRPS-regulated operons in *E. coli* such as the *papA-I* and *unc* operons were also found under such regulation in *C. cellulolyticum*. Third, in *C. cellulolyticum*, stem-loop structures were found near the majority of 3′ iPSs but not 5′ iPSs, which appears consistent with the presence of only 3′ to 5′ (but not 5′ to 3′) exoribonuclease activity in *E. coli*.

However, although SRPS appears to be a conserved global regulatory mechanism, Gram-positive and Gram-negative bacteria appear to employ distinct molecular machineries for SRPS, that is, the endoribonucleases (for example, RNase E in *E. coli*; RNase Y and J in *B. subtilis*) and the exoribonucleases (for example, 3′ to 5′ exoribonucleases in *E. coli*; both 3′ to 5′ and 5′ to 3′ exoribonucleases in *B. subtilis*). *C. cellulolyticum* harbours the typical Gram-positive ribonuclease genes Ccel_0605 and Ccel_1772, which are predicted to encode RNase Y and J, respectively, based on protein sequence homology. However, homologues of RNase Y and J are absent in *E. coli*^19^.

Our work suggests that the differential transcription of genes in SRPS-controlled operons is underlain by the secondary structures at the termini of processed transcripts. Although protection of RNA by stem-loop structures from exoribonucleases was reported for individual genes^14^,^47^, it was unclear whether and how the profile, position and nature of such genomic elements regulate transcriptome structure and function. Here we showed that the stem-loops are a whole-genome mechanism regulating the TA of genes encoded on polycistronic mRNA, and moreover, the degree of protection is correlated with Δ*G* of stem-loops located at their termini on a genome-wide scale. The Δ*G* of RNA secondary structures located at 3′ termini of processed transcripts was lower than those at 5′ termini, suggesting stronger protection of the 3′ termini. This bias of 3′-end protection might be linked to the observation that both 3′ to 5′ exoribonucleases (PNPase and RNase R^19^, encoded by Ccel_1707 and Ccel_2247, respectively) and 5′ to 3′ exoribonucleases (RNase J^48^, encoded by Ccel_1772) were present in *C. cellulolyticum*, where the former exhibit at least 70% higher transcript level than RNase J (Supplementary Data 1). However, not all processed transcripts are associated with stem-loops; in particular, certain high-abundance processed transcripts are free of stem-loops, suggesting presence of certain *trans*-acting factors (for example, Hfq^49^ and sRNAs^50^) that block RNase action.

Although processing of the *cip-cel* transcript was proposed previously employing northern blot and primer extension^51^, the existence of internal promoters cannot be excluded because of the inability to distinguish between TS and PS. Here we revealed that SRPS mechanism is a layer of transcriptional regulation for *cip-cel* operon that precisely determines the organism-specific recipe of cellulosomes. The other large cellulosomal cluster in *C. cellulolyticum*, called *xyl-doc* (Ccel_1229-1242; encoding 14 cellulosomal hemicellulases) and not transcribed under the culture conditions tested, is predicted to be regulated in a similar manner, as it is transcribed as a single operon^52^ and harbours five stem-loop structures in its intergenic regions (Supplementary Fig. 4b). These findings, together with our previously reported ‘Cellulosome Degradome' of *C. cellulolyticum*^26^, allow us to propose a two-tiered mechanism for the global regulation of cellulolysis. Tier I, which consists of Carbon Catabolite Repression (mainly targeting cellulose-degrading enzymes) and two-component systems (primarily for hemicellulases), operates at the inter-operon level and controls differential transcription among cellulolytic operons^26^. Tier II, enabled by SRPS, functions at the intra-operon level and regulates the relative abundance of core cellulosomal subunits encoded within individual operons. Under the carbon substrates tested in this study, Tier I appears to underlie the ‘transience' (that is, being substrate-specific) of the cellulosome as a complex, as suggested by the sensitivity of transcription of Tier I genes to changes in carbon substrates^26^. On the other hand, Tier II might underlie the ‘permanency' (that is, being substrate-independent, yet organism-specific) of the cellulosome, as the operon organization and the substrate-independent transcript ratio of the intra-operon genes precisely determine the relative amount of the core cellulosomal subunits encoded by the *cip-cel* cluster. Therefore, the genome-wide recipe of the Cellulose Degradome is hierarchically regulated: mobilization of a particular cellulolytic module is sensitive to specific carbon substrates at the transcriptional level, yet composition and stoichiometry within a module are precisely determined at the post-transcriptional level. The resulted recipes of cellulosomes are likely organism-specific and crucial to the respective strategy of adaptation and competition in individual cellulolytic microbes, as cellulose is frequently the only carbon sources available to them.

RNA processing was thought to be sensitive to a particular nutrient environment. Certain ligands can bind to the folding structure of 5′ leader of mRNA (known as riboswitch) to induce or inhibit RNA processing, such as the induction of the *trp* operon by tryptophan^53^ and the inhibition of *thrS* by threonine^54^ in *B. subtilis*. However, RNA processing of *cip-cel* appears to be independent from the various carbon sources tested and instead directed by intrinsic, genome-encoded signals (that is, the intergenic stem-loops). Such a distinct regulatory manner can also benefit the organism, as for many protein complexes optimal functioning *in vivo* would require a recipe that is relatively inert to environment changes.

The conservation and species-specificity of PS and stem-loops on the various cellulosome-producing *Clostridium* spp. genomes suggested evolution of cellulosome (and likely a much broader spectrum of protein complexes) is shaped by the number, position and nature of such genomic elements. During evolution of operons, this RNA-mediated mechanism might run in parallel or be linked to variation of diversity, origin or copy number of protein-coding genes, such as the numerous glycosyl hydrolase genes in polysaccharide-degrading loci of both Gram-positive (for example, *Clostridium* spp.^25^) and Gram-negative bacteria (for example, *Bacteroides* spp.^55^). This previously unknown driving force, further powered by combinatorial employment of these regulatory elements (such as that witnessed in *cip-cel*), should have helped creating the diversity and specialization of protein machineries in *Clostridium* spp. and other life-forms on our planet.

On the other hand, the association of iPSs with 120 *C. cellulolyticum* genes, which are from a wide spectrum of functional categories points to a general strategy to specify the stoichiometry of protein complexes and functionally related proteins *in vivo*. The key regulators discovered here, such as the stem-loops whose strength of promoting transcript-abundance is encoded by the primary sequence alone, suggest a succinct, efficient and readily adoptable approach to specify and tune the relative TA of genes within a single operon. Thus, they might find applications in design and engineering of protein-machineries, both *in vitro* and *in vivo*.

## Methods

### Strains and culture conditions

*C. cellulolyticum* ATCC35319 (H10) was cultured anaerobically in 250 ml flasks with 100 ml working volume of DCB-1 medium at 35 °C. The carbon substrates of 3.0 g l^−1^ glucose, 3.0 g l^−1^ cellobiose or 5 g l^−1^ cellulose were used in replicated cultures. A 1% (v/v) culture pre-adapted on various substrates in vials was used for inoculation.

### Pairs of Hyb and Exo libraries

Mapping of TSs and PSs requires the ability to discriminate between primary and processed transcripts^56^. The dRNA-Seq approach accomplishes the discrimination by sequencing pairs of differential cDNA libraries: one library (denoted **Hyb**) from all primary and secondary mRNA (enriched using a subtractive hybridization), and the other (denoted **Exo**) selecting for only primary transcripts (by terminator exonuclease treatment that degrades 5′P but not 5′PPP RNA)^56^. The primary and processed transcripts are distinguished by comparison between results from the two methods, as a transcript that has undergone processing would be characterized by a much lower level of transcript under Exo than under Hyb, whereas a transcript without undergoing processing would feature equivalent transcript level between the two libraries. In addition, locations of the start of a primary transcript are indicated by an elevated and sharp 5′ flank under both Exo and Hyb, whereas the post-transcriptional processed sites are indicated by an elevated and sharp 5′ flank under Hyb yet a much lower transcript level in the downstream flanking regions under Exo.

Total RNA was isolated from *C. cellulolyticum* cultures harvested at the mid-log phase using RNeasy Mini Kit (Qiagen). Genomic DNA was removed by RNase-Free DNase Set (Qiagen). RNA quality was determined using Bioanalyser (Agilent) and quantified using ND-2000 (NanoDrop Technologies).

Two strategies were employed for purification of mRNA from total RNA. (i) The Hyb strategy removed 16S and 23S rRNA from total RNA using MicrobExpress Bacterial mRNA Purification kit (Ambion) according to the manufacturer's protocol with the exception that no more than 5 μg total RNA was treated per enrichment reaction. (ii) The other Exo strategy depleted the rRNA in total RNA via treatment with Terminator 5′-phosphate-dependent exonuclease (Epicentre) for 60 min at 30 °C. One unit of Terminator 5′-phosphate-dependent exonuclease per 1 μg total RNA was used. Following organic extraction (25:24:1 (v/v) phenol/chloroform/isoamyalcohol), the enriched mRNA was precipitated overnight with 2.5 volumes of an mixture of ethanol and 0.1 M sodium acetate (pH 6.5).

Depletion of 16S and 23S rRNA was confirmed by 2100 Bioanalzyer (Agilent) and gel electrophoresis before preparation of cDNA fragment libraries. RNA was reversely transcribed using random primers (Invitrogen) and Superscript III (Invitrogen). The resulted cDNA libraries were sequenced with 2 × 100-bp paired-end runs using Illumina HiSeq 2000 sequencer.

### Mapping reads to the genome

A customized computational pipeline was developed. It starts with a quality control step that removed low-quality bases located at the end of each read. Then high-quality sequencing reads were mapped to the *C. cellulolyticum* genome (GenBank: NC_011898) with SOAP^57^ (the mismatch number parameter (-v) as 2); reads that did not align uniquely to the genome or were mapped to rRNA were discarded. The transcript level of an annotated gene was quantified based on trimmed mean of *M* values via the software edgeR^58^.

### Annotation of TSs and PSs

The genome-wide TS and PS maps for *C. cellulolyticum* were annotated based on differential enrichment of reads in RNA ends (ESs) in Exo versus Hyb libraries in two biological replicates under each of the three culture conditions of glucose, cellobiose and cellulose. The annotation procedure mainly includes four steps: (i) determination of reads-start windows on the genome; (ii) identification of ES windows from all reads-start windows, (iii) calculation of the number of reads that start within a given ES window from the paired Exo and Hyb libraries, respectively; (iv) distinguishing TSs and PSs from the ESs, based on differential enrichment of reads in the ES windows from an Exo library as compared with from its paired Hyb library; (v) producing the final lists of TSs and PSs. These steps were detailed in Supplementary Methods.

### Annotation of operons and their polarity

The operons were manually annotated by combining information from four sources: (i) the regions with a continuous coverage of mapped reads; (ii) the structural integrity of polycistronic mRNA as indicated by paired-end reads; (iii) the 686 putative *C. cellulolyticum* operons as predicted by DOOR^30^ and (iv) the TS map.

The polarity of each operon was analysed by comparing the TA of consecutive genes within the operon, which classified the operons into four patterns. (i) ‘Down' pattern: the operons show natural polarity, where transcription levels of the genes progressively decrease towards the end of the operon (log_2_(TA_X_/TA_Y_)>0.5, where TA_X_ is the TA of gene X, which is proximal to the promoter and TA_Y_ refers to the gene Y, which is consecutive and downstream of gene X); (ii) ‘Flat' pattern: the expression levels of genes in an operon were approximately equimolar (−0.5≤log_2_(TA_X_/TA_Y_)≤0.5); (iii) ‘Up' pattern: the operons show reversed natural polarity, where the transcription levels of the genes progressively increase towards the end of the operon (log_2_(TA_X_/TA_Y_)<−0.5); (iv) ‘Complex' pattern: those operons that feature nonlinear relationship between the order of a gene and the transcript level of the gene.

### Functional enrichment analysis

The statistical significance of enrichment of each PS category (iPS, cPS or uPS) in the four types of operon patterns (Down, Flat, Up and Complex types of operons) was calculated as follows: for example, let ‘*N*' be the total number of operons harbouring PSs, ‘*n*' be the number of iPS-harbouring operons, ‘*M*' be the total number of PS-harbouring operons assigned to a operon pattern (such as ‘complex') and ‘*m*' be the number of iPS-harbouring operons assigned to this operon pattern. The *P*-value was estimated for enrichment of iPS harbouring operon in a operon pattern based on the hypergeometric test:


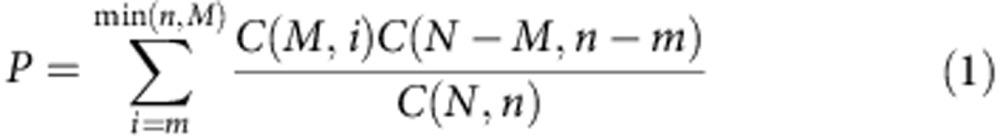


in which *C*(*x,y*) is the combinational number of choosing *y* items out of *x* items. Enrichment of COG-slim terms with *P* value≤0.05 was considered as statistically significant.

### RNA secondary structure prediction

Sequences of 100-nt long that are neighbouring PSs or non-PS intergenic region (as the control) were used for the prediction of RNA secondary structure. For a given PS, the flanking sequence of the PS was extracted, which included the 49 nt region upstream of the PS and the 50 nt region downstream of the PS. As the control sequences, non-PS intergenic region within PS-harbouring operons were extracted. For a given non-PS intergenic region, if its original length is lower than 100 nt, the sequence was extended to 100 nt in length, with 50 nt flanking the centre position of the sequence at each of the two ends; if its original length is over 100 nt, then only 100 nt of the sequence was kept, with 50 nt flanking the centre position of the sequence at each of the two ends.

As any structure of transcript termini, irrespective of its shape, might potentially protect the RNA from degradation^16^, the full RNA secondary structure of the sequences as defined above was computed using Mfold with default parameters^32^. The minimum of folding free energy (Δ*G*) of the sequences was used for further statistical analysis that compared the values and distribution of Δ*G* between the two types of sequences as defined above. Furthermore, to probe the potential role of stem-loops specifically, single-stem-loop structures of those sequences associated with iPSs were also predicted and visualized using Mfold, which were allowed to harbour no more than five mispairs within three places and were required to have Δ*G* that were lower than −5.

### Northern blot hybridization

Two micrograms of total RNA were isolated from bacterial cultures on cellobiose. The sample was analysed via electrophoreses on 1% agarose-formaldehyde gel and blotted onto a positively charged nylon membrane (Roche) using NorthernMax kit (Life Technologies). DIG-labelled DNA probes for the detection of specific transcripts were generated with a DIG labelling and detection kit (Roche) as described by the manufacturer's instructions using the oligonucleotides listed in Supplementary Data 7.

### Quantitative reverse transcription–PCR (qRT–PCR)

To validate the mRNA-Seq-based transcript quantification of genes from the *cip-cel* operon, we measured the absolute transcript copy number via qRT–PCR. The qRT–PCR was performed using the SYBR Green I on LightCycler480II using FastStart Universal SYBR Green Master (Roche) and the results normalized via abundance of 16S rRNA. The primer sets for qRT–PCR were listed in Supplementary Data 7.

### Functional analysis of iPSs and stem-loops

Intergenic regions from the *cip-cel* operon were amplified, respectively, by PCR using synthetic oligonucleotide primers (listed in Supplementary Data 7). To probe their promoter activity, the PCR products of various intergenic regions were digested with *Pst*I and *Mlu*I. The digested fragments, respectively, inserted into the *C. cellulolyticum*–*E. coli* shuttle vector pMTC6, which harbours a reporter gene *fbfp* (encoding a green fluorescence protein) coupled to the *pthl* promoter (thiolase gene promoter from *C. acetobutylicum*)^59^, resulting in the substitution of the original *pthl* promoter by the various intergenic regions from the *cip-cel* operon.

To probe the functional role of the stem-loop structure harboured by the intergenic regions from the *cip-cel* operon, a dual fluorescence reporter system was constructed by inserting the gene *mcherry* (encoding a red fluorescence protein) after *fbfp* gene in pMTC6 using *Eco*RI and *Bam*HI. In the resulted plasmid, the green-fluorescence-encoding *fbfp* and the red-fluorescence-encoding *mcherry* were expressed in a single operon, with a *Bgl*II restriction site between the two genes for introduction of a stem-loop harbouring intergenic region (Fig. 6a).

The recombinant plasmids were methylated *in vitro* with *Msp*I methyltransferase before electro-transformation of *C. cellulolyticum*^60^. The promoter activity of intergenic regions was determined via fluorescence intensity of FbFP expressed in *C. cellulolyticum* transformants as compared with the control. The degree of protection of stem-loop structure conveyed to the transcript of the neighbouring genes (in this case, the downstream gene) was quantified by comparing the fluorescence intensity of FbFP and mCherry. The averages of three biological replicates were shown.

### Analysis of mutant strains

Plasmids for targeted disruption of *C. cellulolyticum* genes were constructed from Targetron plasmid pSY6 (ref. 61). Targeting sites for disruption and the intron retargeting primers were designed with tools on http://clostron.com/ (Supplementary Data 7)^62^. The 353-bp intron targeting regions were prepared by overlap extension (SOE) PCR method using above primers and then inserted into pSY6 for targeted disruption of two genes in the *cip-cel* operon: *cipC* (mutant strain designated as ΔcipC) and *orfX* (ΔorfX). The recombinant plasmids were methylated and transformed into *C. cellulolyticum*. The mutants were validated by colony PCRs (Supplementary Data 7). Positive colonies were inoculated into fresh medium supplemented with erythromycin. Transcript level of genes in the *cip-cel* cluster (Ccel_0728-0740) in mutants grown on cellobiose and cellulose was determined by qRT–PCR as described. The wild-type strain was used as a control in all experiments.

### Phylogenetic analysis of cellulosomal loci in Clostridia

Cellulosome clusters from these six additional Clostridia species (*C. papyrosolvens*, *C. sp*. BNL1100, *C. josui*, *C. termitidis*, *C. acetobutylicum* and *C. cellulovorans*) were identified by BLAST with *e*-value cutoff of 1e-5, using the *cip-cel* operon from *C. cellulolyticum* as query. Organismal phylogeny of these seven species was derived via Maximum Likelihood methods using 16S rRNA sequences. All positions containing gaps and missing data were eliminated, which resulted in a total of 1,382 positions in the final multiple-sequence alignment. Phylogenetic analyses of the *Clostridium* spp. were conducted in MEGA5 (ref. 63).

## Author contributions

J.X., C.X. and R.H. designed the experiments; C.X., R.H., L.T., X.J. and Y.W. performed the experiments; G.C. and Q.C. contributed two of the *C. cellulolyticum* mutants (H10ΔcipC and H10ΔorfX); C.X., R.H., J.H. and J.X. analysed data; C.X. and J.X. wrote the paper.

## Additional information

**Accession codes:** The mRNA-Seq data from this article was deposited in the National Center for Biotechnology Information Gene Expression Omnibus (GEO) under accession number GSE57652.

**How to cite this article:** Xu, C. *et al*. Cellulosome stoichiometry in *Clostridium cellulolyticum* is regulated by selective RNA processing and stabilization. *Nat. Commun*. 6:6900 doi: 10.1038/ncomms7900 (2015).

## Supplementary Material

Supplementary Figures, Supplementary Tables, Supplementary Methods and Supplementary References Supplementary Figures 1-4, Supplementary Tables 1-2, Supplementary Methods and Supplementary References

Supplementary Data 1 The transcript level of genes under various carbon substrates in Clostridium cellulolyticum. The transcript abundance of each gene under a particular condition was determined based on trimmed mean of M values (TMM) via the software edgeR (Methods).

Supplementary Data 2 Maps of TS on the Clostridium cellulolyticum genome. The ratio of reads between Exo and Hyb libraries under three carbon sources were respectively listed, in which NA means “not available”. The TSs were grouped into three categories based on their genomic context: (i) iTSs (i.e., “intergenic” TSs), which are located at intergenic region; (ii) cTSs (i.e., “coding-region” TSs), which are within an annotated CDS on the same strand; (iii) oTSs (i.e., “orphan” TSs), which are orphans with no annotated genes in their proximity.

Supplementary Data 3 Maps of PS on the Clostridium cellulolyticum genome. The ratio of reads between Exo and Hyb libraries under three carbon sources were respectively listed, in which NA means "not available”. Based on their physical positions relative to the coding regions in an operon, the PSs can be classified into three categories: (i) cPSs (59 in total), which are positioned internally within annotated CDS. (ii) uPSs (24 in total), which are located at the 5′ UTR of operons; (iii) iPSs (72 in total), which are located at intergenic regions and can potentially guide cleavages that physically uncouple genes encoded on a single polycistronic mRNA. For each of the PSs, its associated genes were also shown. Furthermore, the PS-flanking sequences and their RNA folding energy (ΔG; kcal mol-1) were presented.

Supplementary Data 4 The experimentally determined operon map of Clostridium cellulolyticum. The TSs, numbers of operon-harboring PSs and operon transcriptional polarities (up, down, flat, complex or unknown) were listed.

Supplementary Data 5 Sequences of the non-iPS intergenic regions within PS-harboring operons.

Supplementary Data 6 Stem-loop structures identified in this study that potentially regulate protein-complex stoichiometry. In Ccel, the stem-loop structures flanking iPSs and their free energy (ΔG) were listed in the first sheet. In the second sheet, the stem-loop structures in the cip-cel-like clusters from the seven Clostridium spp. of C. cellulovorans (Cloc), C. acetobutylicum (Cace), C.papyrosolvens (Cpap), C.josui (Cjos), C. sp. BNL1100 (Cbnl), C. termitidis (Cter) and C. cellulolyticum (Ccel) were listed.

Supplementary Data 7 The list of PCR primer sets used in this study.

## References

[b1] Felix C. R. & Ljungdahl L. G. The cellulosome: the exocellular organelle of Clostridium. Annu. Rev. Microbiol. 47, 791–819 (1993). 8257116 10.1146/annurev.mi.47.100193.004043

[b2] Pakrasi H. B. Genetic analysis of the form and function of photosystem I and photosystem II. Annu. Rev. Genet. 29, 755–776 (1995). 8825493 10.1146/annurev.ge.29.120195.003543

[b3] White M. M. Pretty subunits all in a row: using concatenated subunit constructs to force the expression of receptors with defined subunit stoichiometry and spatial arrangement. Mol. Pharmacol. 69, 407–410 (2006). 16293710 10.1124/mol.105.020727

[b4] Rochat T., Bouloc P. & Repoila F. Gene expression control by selective RNA processing and stabilization in bacteria. FEMS Microbiol. Lett. 344, 104–113 (2013). 23617839 10.1111/1574-6968.12162

[b5] Nilsson P. & Uhlin B. E. Differential decay of a polycistronic *Escherichia coli* transcript is initiated by RNaseE-dependent endonucleolytic processing. Mol. Microbiol. 5, 1791–1799 (1991). 1943710 10.1111/j.1365-2958.1991.tb01928.x

[b6] Newbury S. F., Smith N. H. & Higgins C. F. Differential mRNA stability controls relative gene expression within a polycistronic operon. Cell 51, 1131–1143 (1987). 2446776 10.1016/0092-8674(87)90599-x

[b7] Jordi B. J. A. M., Dencamp I. E. L. O., Dehaan L. A. M., Vanderzeijst B. A. M. & Gaastra W. Differential decay of RNA of the CFA/I fimbrial operon and control of relative gene expression. J. Bacteriol. 175, 7976–7981 (1993). 7504669 10.1128/jb.175.24.7976-7981.1993 PMC206977

[b8] Patel A. M. & Dunn S. D. RNase E-dependent cleavages in the 5′ and 3′ regions of the *Escherichia coli* unc mRNA. J. Bacteriol. 174, 3541–3548 (1992). 1534325 10.1128/jb.174.11.3541-3548.1992 PMC206039

[b9] Owolabi J. B. & Rosen B. P. Differential mRNA stability controls relative gene expression within the plasmid-encoded arsenical resistance operon. J. Bacteriol. 172, 2367–2371 (1990). 2185215 10.1128/jb.172.5.2367-2371.1990 PMC208871

[b10] Cam K., Rome G., Krisch H. M. & Bouche J. P. RNase E processing of essential cell division genes mRNA in *Escherichia coli*. Nucleic Acids Res. 24, 3065–3070 (1996). 8760895 10.1093/nar/24.15.3065 PMC146031

[b11] Lodato P. B. & Kaper J. B. Post-transcriptional processing of the LEE4 operon in enterohaemorrhagic *Escherichia coli*. Mol. Microbiol. 71, 273–290 (2009). 19019141 10.1111/j.1365-2958.2008.06530.x PMC2782684

[b12] Belasco J. G., Beatty J. T., Adams C. W., von Gabain A. & Cohen S. N. Differential expression of photosynthesis genes in *R. capsulata* results from segmental differences in stability within the polycistronic rxcA transcript. Cell 40, 171–181 (1985). 2981627 10.1016/0092-8674(85)90320-4

[b13] Klug G. The role of mRNA degradation in the regulated expression of bacterial photosynthesis genes. Mol. Microbiol. 9, 1–7 (1993). 7692215 10.1111/j.1365-2958.1993.tb01663.x

[b14] McDowall K. J., Kaberdin V. R., Wu S. W., Cohen S. N. & Lin-Chao S. Site-specific RNase E cleavage of oligonucleotides and inhibition by stem-loops. Nature 374, 287–290 (1995). 7533896 10.1038/374287a0

[b15] Collins J. A., Irnov I., Baker S. & Winkler W. C. Mechanism of mRNA destabilization by the glmS ribozyme. Genes Dev. 21, 3356–3368 (2007). 18079181 10.1101/gad.1605307 PMC2113035

[b16] Pfleger B. F., Pitera D. J., Smolke C. D. & Keasling J. D. Combinatorial engineering of intergenic regions in operons tunes expression of multiple genes. Nature Biotechnol. 24, 1027–1032 (2006). 16845378 10.1038/nbt1226

[b17] Guell M. . Transcriptome complexity in a genome-reduced bacterium. Science 326, 1268–1271 (2009). 19965477 10.1126/science.1176951

[b18] Mitschke J., Vioque A., Haas F., Hess W. R. & Muro-Pastor A. M. Dynamics of transcriptional start site selection during nitrogen stress-induced cell differentiation in Anabaena sp. PCC7120. Proc. Natl Acad. Sci. USA 108, 20130–20135 (2011). 22135468 10.1073/pnas.1112724108 PMC3250118

[b19] Arraiano C. M. . The critical role of RNA processing and degradation in the control of gene expression. FEMS Microbiol. Rev. 34, 883–923 (2010). 20659169 10.1111/j.1574-6976.2010.00242.x

[b20] Ludwig H. . Transcription of glycolytic genes and operons in Bacillus subtilis: evidence for the presence of multiple levels of control of the gapA operon. Mol. Microbiol. 41, 409–422 (2001). 11489127 10.1046/j.1365-2958.2001.02523.x

[b21] Desvaux M. *Clostridium cellulolyticum*: model organism of mesophilic cellulolytic clostridia. FEMS Microbiol. Rev. 29, 741–764 (2005). 16102601 10.1016/j.femsre.2004.11.003

[b22] Doi R. H., Kosugi A., Murashima K., Tamaru Y. & Han S. O. Cellulosomes from mesophilic bacteria. J. Bacteriol. 185, 5907–5914 (2003). 14526000 10.1128/JB.185.20.5907-5914.2003 PMC225047

[b23] Fendri I. . The cellulosomes from *Clostridium cellulolyticum*: identification of new components and synergies between complexes. FEBS J. 276, 3076–3086 (2009). 19490109 10.1111/j.1742-4658.2009.07025.x

[b24] Bayer E. A., Belaich J. P., Shoham Y. & Lamed R. The cellulosomes: multienzyme machines for degradation of plant cell wall polysaccharides. Annu. Rev. Microbiol. 58, 521–554 (2004). 15487947 10.1146/annurev.micro.57.030502.091022

[b25] Bayer E. A., Lamed R., White B. A. & Flint H. J. From cellulosomes to cellulosomics. Chem Rec. 8, 364–377 (2008). 19107866 10.1002/tcr.20160

[b26] Xu C. . Structure and regulation of the cellulose degradome in *Clostridium cellulolyticum*. Biotechnol. Biofuels 6, 73 (2013). 23657055 10.1186/1754-6834-6-73 PMC3656788

[b27] Hemme C. L. . Sequencing of multiple clostridial genomes related to biomass conversion and biofuel production. J. Bacteriol. 192, 6494–6496 (2010). 20889752 10.1128/JB.01064-10 PMC3008519

[b28] Fendri I. . Regulation of cel genes of *C. cellulolyticum*: identification of GlyR2, a transcriptional regulator regulating cel5D gene expression. PLoS One 8, e44708 (2013). 23349658 10.1371/journal.pone.0044708 PMC3551867

[b29] Tatusov R. L., Galperin M. Y., Natale D. A. & Koonin E. V. The COG database: a tool for genome-scale analysis of protein functions and evolution. Nucleic Acids Res. 28, 33–36 (2000). 10592175 10.1093/nar/28.1.33 PMC102395

[b30] Mao F. L., Dam P., Chou J., Olman V. & Xu Y. DOOR: a database for prokaryotic operons. Nucleic Acids Res. 37, D459–D463 (2009). 18988623 10.1093/nar/gkn757 PMC2686520

[b31] Deckers-Hebestreit G. & Altendorf K. The Fo complex of the proton-translocating F-type ATPase of *Escherichia coli*. J. Exp. Biol. 172, 451–459 (1992). 1337099 10.1242/jeb.172.1.451

[b32] Zuker M. Mfold web server for nucleic acid folding and hybridization prediction. Nucleic Acids Res. 31, 3406–3415 (2003). 12824337 10.1093/nar/gkg595 PMC169194

[b33] Santangelo T. J. & Artsimovitch I. Termination and antitermination: RNA polymerase runs a stop sign. Nat. Rev. Microbiol. 9, 319–329 (2011). 21478900 10.1038/nrmicro2560 PMC3125153

[b34] Kingsford C. L., Ayanbule K. & Salzberg S. L. Rapid, accurate, computational discovery of Rho-independent transcription terminators illuminates their relationship to DNA uptake. Genome Biol. 8, R22 (2007). 17313685 10.1186/gb-2007-8-2-r22 PMC1852404

[b35] Desvaux M. The cellulosome of *Clostridium cellulolyticum*. Enzyme Microb. Technol. 37, 373–385 (2005).

[b36] Blouzard J. C. . Modulation of cellulosome composition in *Clostridium cellulolyticum*: adaptation to the polysaccharide environment revealed by proteomic and carbohydrate-active enzyme analyses. Proteomics 10, 541–554 (2010). 20013800 10.1002/pmic.200900311

[b37] Maamar H. . Cellulolysis is severely affected in *Clostridium cellulolyticum* strain cipCMut1. Mol. Microbiol. 51, 589–598 (2004). 14756796 10.1046/j.1365-2958.2003.03859.x

[b38] Cui G. Z. . Improvement of ClosTron for successive gene disruption in *Clostridium cellulolyticum* using a pyrF-based screening system. Appl. Microbiol. Biotechnol. 98, 313–323 (2014). 24190496 10.1007/s00253-013-5330-y

[b39] Lehnik-Habrink M., Lewis R. J., Mader U. & Stulke J. RNA degradation in *Bacillus subtilis*: an interplay of essential endo- and exoribonucleases. Mol. Microbiol. 84, 1005–1017 (2012). 22568516 10.1111/j.1365-2958.2012.08072.x

[b40] Han S. O., Yukawa H., Inui M. & Doi R. H. Transcription of *Clostridium cellulovorans* cellulosomal cellulase and hemicellulase genes. J. Bacteriol. 185, 2520–2527 (2003). 12670976 10.1128/JB.185.8.2520-2527.2003 PMC152600

[b41] Fontes C. M. & Gilbert H. J. Cellulosomes: highly efficient nanomachines designed to deconstruct plant cell wall complex carbohydrates. Annu. Rev. Biochem. 79, 655–681 (2010). 20373916 10.1146/annurev-biochem-091208-085603

[b42] Sabathe F., Belaich A. & Soucaille P. Characterization of the cellulolytic complex (cellulosome) of *Clostridium acetobutylicum*. FEMS Microbiol. Lett. 217, 15–22 (2002). 12445640 10.1111/j.1574-6968.2002.tb11450.x

[b43] Belasco J. G. All things must pass: contrasts and commonalities in eukaryotic and bacterial mRNA decay. Nat. Rev. Mol. Cell Biol. 11, 467–478 (2010). 20520623 10.1038/nrm2917 PMC3145457

[b44] Bernstein J. A., Khodursky A. B., Lin P. H., Lin-Chao S. & Cohen S. N. Global analysis of mRNA decay and abundance in Escherichia coli at single-gene resolution using two-color fluorescent DNA microarrays. Proc. Natl Acad. Sci. USA 99, 9697–9702 (2002). 12119387 10.1073/pnas.112318199 PMC124983

[b45] Steglich C. . Short RNA half-lives in the slow-growing marine cyanobacterium *Prochlorococcus*. Genome Biol. 11, R54 (2010). 20482874 10.1186/gb-2010-11-5-r54 PMC2897979

[b46] Dressaire C. . Role of mRNA stability during bacterial adaptation. PLoS One 8, e59059 (2013). 23516597 10.1371/journal.pone.0059059 PMC3596320

[b47] Emory S. A., Bouvet P. & Belasco J. G. A 5′-terminal stem-loop structure can stabilize mRNA in *Escherichia coli*. Genes Dev. 6, 135–148 (1992). 1370426 10.1101/gad.6.1.135

[b48] Commichau F. M. . Novel activities of glycolytic enzymes in Bacillus subtilis: interactions with essential proteins involved in mRNA processing. Mol. Cell Proteomics 8, 1350–1360 (2009). 19193632 10.1074/mcp.M800546-MCP200 PMC2690492

[b49] Vogel J. & Luisi B. F. Hfq and its constellation of RNA. Nat. Rev. Microbiol. 9, 578–589 (2011). 21760622 10.1038/nrmicro2615 PMC4615618

[b50] Wagner E. G. Kill the messenger: bacterial antisense RNA promotes mRNA decay. Nature Struct. Mol. Biol. 16, 804–806 (2009). 19654618 10.1038/nsmb0809-804

[b51] Maamar H., Abdou L., Boileau C., Valette O. & Tardif C. Transcriptional analysis of the cip-cel gene cluster from *Clostridium cellulolyticum*. J. Bacteriol. 188, 2614–2624 (2006). 16547049 10.1128/JB.188.7.2614-2624.2006 PMC1428388

[b52] Celik H. . A two-component system (XydS/R) controls the expression of genes encoding CBM6-containing proteins in response to straw in *Clostridium cellulolyticum*. PLoS ONE 8, e56063 (2013). 23418511 10.1371/journal.pone.0056063 PMC3572039

[b53] Winkler W. C., Nahvi A., Roth A., Collins J. A. & Breaker R. R. Control of gene expression by a natural metabolite-responsive ribozyme. Nature 428, 281–286 (2004). 15029187 10.1038/nature02362

[b54] Condon C., Putzer H. & Grunberg-Manago M. Processing of the leader mRNA plays a major role in the induction of thrS expression following threonine starvation in *Bacillus subtilis*. Proc. Natl Acad. Sci. USA 93, 6992–6997 (1996). 8692931 10.1073/pnas.93.14.6992 PMC38922

[b55] Xu J. . Evolution of symbiotic bacteria in the distal human intestine. PLoS Biol. 5, e156 (2007). 17579514 10.1371/journal.pbio.0050156 PMC1892571

[b56] Sharma C. M. . The primary transcriptome of the major human pathogen *Helicobacter pylori*. Nature 464, 250–255 (2010). 20164839 10.1038/nature08756

[b57] Li R. . SOAP2: an improved ultrafast tool for short read alignment. Bioinformatics 25, 1966–1967 (2009). 19497933 10.1093/bioinformatics/btp336

[b58] Robinson M. D. & Oshlack A. A scaling normalization method for differential expression analysis of RNA-seq data. Genome Biol. 11, R25 (2010). 20196867 10.1186/gb-2010-11-3-r25 PMC2864565

[b59] Cui G. Z. . Targeted gene engineering in *Clostridium cellulolyticum* H10 without methylation. J. Microbiol. Methods 89, 201–208 (2012). 22450138 10.1016/j.mimet.2012.02.015

[b60] Jennert K. C. B., Tardif C., Young D. I. & Young M. Gene transfer to *Clostridium cellulolyticum* ATCC 35319. Microbiology 146, 3071–3080 (2000). 11101665 10.1099/00221287-146-12-3071

[b61] Shao L. . Targeted gene disruption by use of a group II intron (targetron) vector in *Clostridium acetobutylicum*. Cell Res. 17, 963–965 (2007). 17971808 10.1038/cr.2007.91

[b62] Perutka J., Wang W. J., Goerlitz D. & Lambowitz A. M. Use of computer-designed group II introns to disrupt *Escherichia coli* DExH/D-box protein and DNA helicase genes. J. Mol. Biol. 336, 421–439 (2004). 14757055 10.1016/j.jmb.2003.12.009

[b63] Tamura K. . MEGA5: molecular evolutionary genetics analysis using maximum likelihood, evolutionary distance, and maximum parsimony methods. Mol. Biol. Evol. 28, 2731–2739 (2011). 21546353 10.1093/molbev/msr121 PMC3203626

