# Supplementary material for: Cellulosome stoichiometry in Clostridium cellulolyticum is regulated by selective RNA processing and stabilization
Source: Nat Commun. 2015 Apr 24;6:6900. doi: 10.1038/ncomms7900 (PMC4423207; doi:10.1038/ncomms7900)
Supplement: Supplementary Figures, Supplementary Tables, Supplementary Methods and Supplementary References — Supplementary Figures 1-4, Supplementary Tables 1-2, Supplementary Methods and Supplementary References [file ncomms7900-s1.pdf]

Supplementary information

Supplementary Figures:

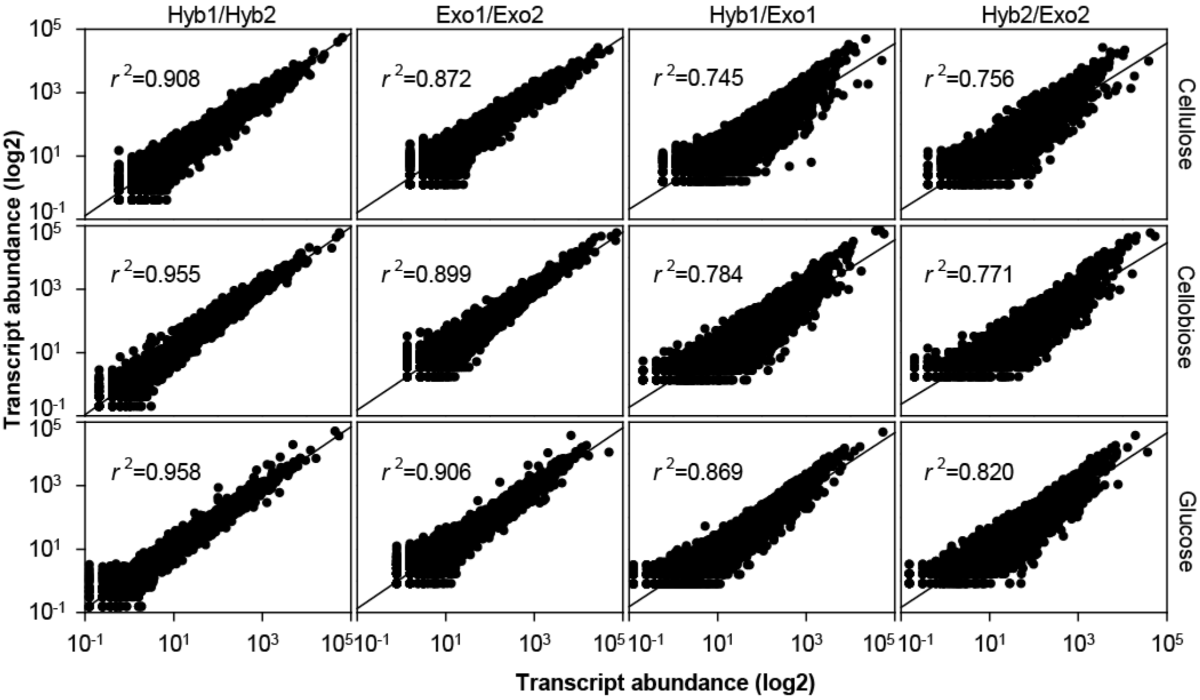

Supplementary Figure 1. Comparison of transcriptomes between Hyb and Exo methods under cellulose, cellobiose and glucose in *Clostridium cellulolyticum*. Each point indicates the abundance of an individual transcript in the biological replicates of Hyb or Exo, or in the two different methods of Hyb and Exo.

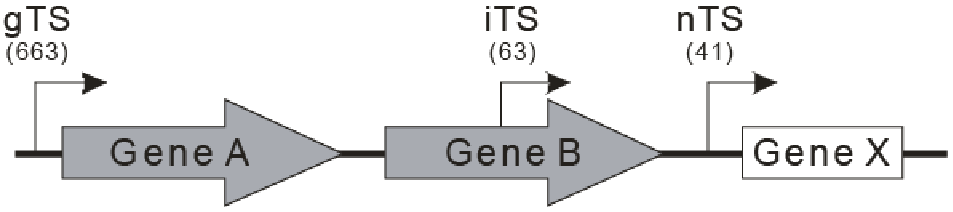

Supplementary Figure 2. Classification of the TSs as predicted by dRNA-Seq. These TSs were grouped into three categories based on their genomic context: (*i*) the 663 gTSs (i.e., “gene” TSs), which are located upstream of a gene; (*ii*) the 63 iTSs (i.e., “internal” TSs), which positioned within a coding sequence; (*iii*) the 41 nTSs (i.e., “non-coding” TSs), which are orphans with no annotated genes in their proximity.

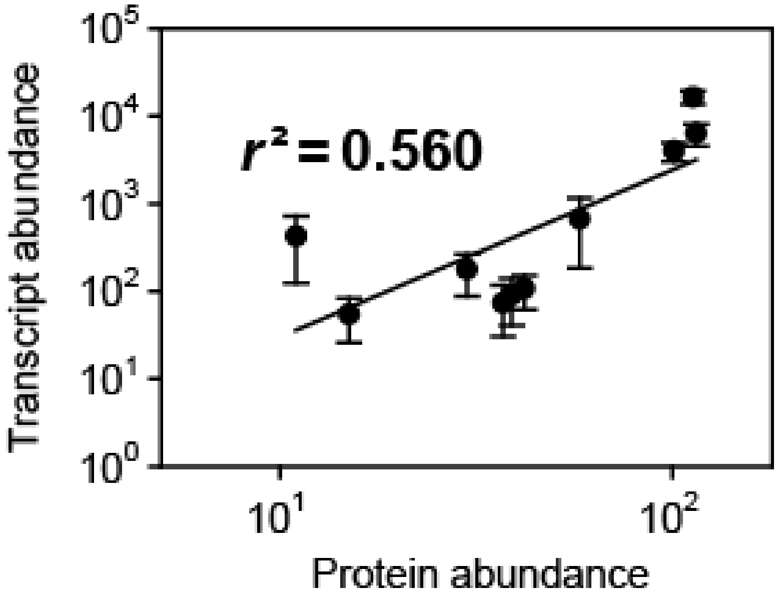

Supplementary Figure 3. Correlation of *cip-cel* gene expression under cellulose between the transcript level and the protein level. The proteome data were derived from reference [1].

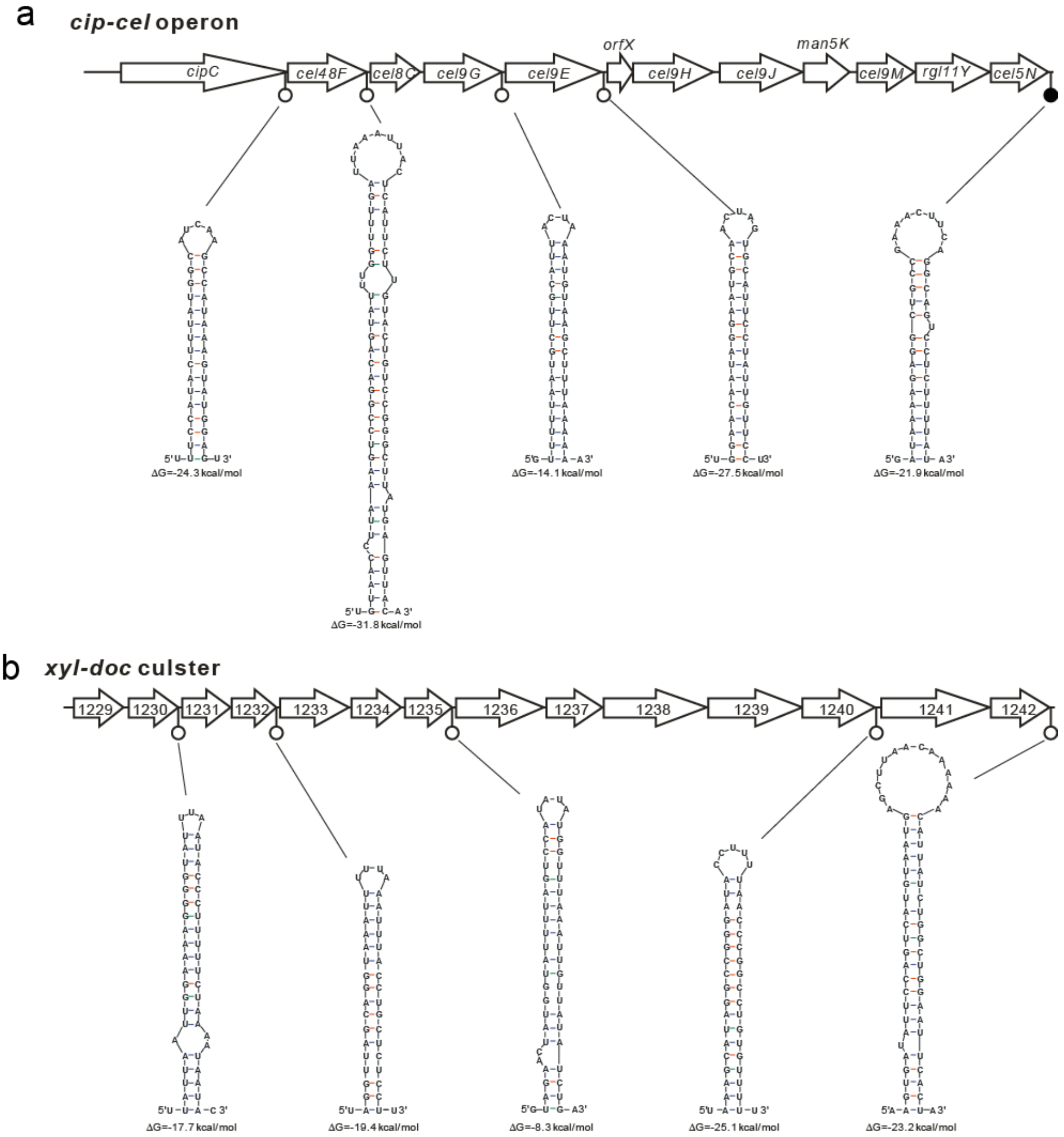

Supplementary Figure 4. Stem-loop structures of the *cip-cel* operon (a) and the *xyl-doc* cluster (b). The stem-loop structures were found at the intergenic regions as specified in the figure.

Supplementary Tables:

Supplementary Table 1. Differential RNA-Seq of *Clostridium cellulolyticum* ATCC35319

cDNA Library[a]	Raw reads	Reads map to genome	Total uniquely mapped reads	Total uniquely mapped reads (no rRNA)	Reads map to CDS regions	Sequencing depth	Annotated genome sites (bp)
Glu1-Hyb	4994452*2	8445019(84.55%)	6282179	6135203	5039589(82.14%)	151.2	3575951
Glu2-Hyb	4158859*2	7020694(84.41%)	4941120	4810779	3928405(81.66%)	118.6	3571569
Glu1-Exo	3367159*2	5386746(79.99%)	1302432	1190602	1024090(86.01%)	29.3	2754818
Glu2-Exo	3134869*2	5055987(80.64%)	1335685	1251207	1045093(83.53%)	30.8	2718541
Ceb1-Hyb	4473446*2	7420891(82.94%)	5243945	5085974	4124915(81.10%)	125.6	3350226
Ceb2-Hyb	4848533*2	8124223(83.78%)	5613973	5456900	4505984(82.57%)	134.5	3365033
Ceb1-Exo	3021131*2	4851111(80.29%)	1263085	1172698	966643(82.43%)	28.8	2386898
Ceb2-Exo	3194766*2	4978452(77.92%)	1122405	1026078	856487(83.47%)	25.2	2260552
Cel1-Hyb	3438306*2	5467069(79.51%)	2049026	1840970	1473813(80.06%)	45.5	3047810
Cel2-Hyb	3553563*2	5710905(80.35%)	2227222	2027728	1779966(87.78%)	50.1	3223812
Cel1-Exo	3406521*2	5353984(78.59%)	1210154	1077217	792222(73.54%)	26.5	2383308
Cel2-Exo	3393191*2	5400472(79.58%)	1137213	1025139	757082(73.85%)	25.2	2524637

[a] Glu, Ceb and Cel respectively represent the growth substrate glucose, cellobiose and cellulose. Two independent RNA collections obtained from three independent cultures on each substrate were analyzed. Each RNA sample was respectively purified by Hyb and Exo methods

Supplementary Table 2. Correlation in transcript level of the twelve *cip-cel* genes among the three carbon sources. Correlation coefficients (r^2) among the three carbon sources and those among the biological replicates within each of the carbon sources were listed in gray tables.

r^2	Cel1	Cel2	Ceb1	Ceb2	Glu1	Glu2
Glu2	0.911	0.868	0.942	0.919	0.933	1
Glu1	0.975	0.959	0.957	0.949	1	
Ceb2	0.965	0.925	0.989	1		
Ceb1	0.971	0.938	1			
Cel2	0.984	1				
Cel1	1					

Supplementary methods:

Annotation of RNA end site (ES), transcriptional start site (TS) and processing site (PS)

(*i*) Determination of reads-start windows on the genome. In order to accommodate the possibility of slightly different mapping coordinates of read-starting position among distinct culture conditions or biological replicates, reads in all the twelve libraries (i.e., both Hyb and Exo; cultured under cellulose, cellobiose or glucose respectively; each with two biological libraries) that were mapped to the *C. cellulolyticum* genome were first pooled. For each of the two genome strands, the mapped reads were binned based on their 5'-end alignment position within a 3nt-wide window[2]. Such "reads-start windows" were extended to accommodate the 5'-end alignment positions of additional reads, until the next window was over 3 nt apart. However, if the size of such a potential window exceeded 200 nt, which is the average length of the pair-end fragments, the extension would stop and the window was set to be 200 nt long. All the reads within such orientation-sensitive, post-"extension" windows were considered as defining (and associated with) one candidate ES, whose orientation was designated as the orientation of the corresponding window. The definition of such "reads-start windows" thus accommodated the possible slight difference in mapping coordinates of read-starting position among distinct culture conditions or biological replicates.

(*ii*) Identification of ES windows from all reads-start windows. The detection of an ES window was based on the localization of RNA-end positions, where a significant number of start transcripts[3]. Thus, a parameter for estimating content of start transcript from a ES window in all transcript through this window was introduced, named "initiation ratio". For ES window (*a, b*), the initiation ratio ("*I*") was calculated as:

$$I = \frac{\sum_{i=a}^{b} max(R_i^+ - R_i^-, 0)}{max(\sum_{i=a-200}^{a} R_i^+, \sum_{i=a-199}^{a+1} R_i^+, \ldots, \sum_{i=b-201}^{b-1} R_i^+, \sum_{i=b-200}^{b} R_i^+)}$$

where R_i^+ and R_i^- are respectively the number of read-starts at each nt *i* of ES window at its predicted direction and the opposite direction. If (R_i^+ - R_i^-) is greater than 0 (i. e, max (R_i^+ - R_i^-, 0)), it indicated that there are (R_i^+ - R_i^-) of initiation transcripts in nt *i*. Therefore, sum of max (R_i^+ - R_i^-, 0) from window (a, b) represents the number of all initiation transcripts in window (a, b). On the other hand, as the average length of the pair-end fragments is 200 nt, sum of R_i^+ within 200-bp upstream of each nt represents all transcripts spanning this nt. Here, for the window (a, b), maximum of the sum from each nt in this window is designated as the number of all transcripts partially or completely spanning window (a, b). Therefore, the initiation ratio *I* is calculated as the ratio of number of initiation transcripts to all transcripts partially or completely

spanning window (a, b). To designate a threshold of *I* that characterized ES (i.e., an RNA-end position) in our procedure, it was adjusted based on the *Helicobacter pylori* dRNA-Seq training data set[4] where 69 TSs were identified via independent experimental approaches. The training data set indicated that the initiation ratio for TSs was over 90%. Given the efficiency of RNA cleavage and possibility of overlap of transcription, thus, we set the threshold of initiation ratio in our procedure as 70% for the final ES windows. Those reads-start windows with an initiation ratio of at least 70% were considered as ES windows (with each of them defining an ES), which would proceed to the analysis below.

(*iii*) Calculation of the number of reads that start within a given ES window from the paired Exo and Hyb libraries respectively. For each of the three culture conditions, the number of reads within a given ES window was cataloged from the Exo and Hyb libraries respectively, and then the enrichment ratio of reads in the ES window as compared to the whole-genome is calculated using the binomial distribution[5]. If the enrichment ratios from the two biological replications from the same culture condition were both lower than 0.05, the ES (i.e., as defined by the ES-window) was considered as an ES under that particular condition. Thus, at this step, a list of ESs was produced for each of the three culture conditions.

(*iv*) Distinguishing TSs and PSs from the ESs. As 5'PPP of the primary transcripts will be enriched in the Exo libraries (in which the 5'P transcripts were already removed), the relative abundance of reads from primary transcripts (i.e., TSs) in Exo will be higher than that in Hyb, whereas the relative abundance of reads from processed transcripts (i.e., PSs) in Exo library would be lower than that in Hyb. Therefore, a threshold hold value should be selected for distinguishing TSs and PSs from the ESs based on the ratio of relative abundance of reads in Exo and Hyb. Setting the ratio at a higher value would lead to higher specificity of TSs, yet result in lower sensitivity of TSs; on the other hand, setting the ratio at a lower value would lead to higher specificity of PSs, yet result in lower sensitivity of PSs. Therefore a strategy was adopted that minimized the risk of underestimating TSs and overestimating PSs. Specifically, for TSs, threshold for the ratio of reads in Exo and Hyb was set to 1.0, and only those ESs with the ratio higher than the threshold under <u>any</u> of the three conditions (glucose, cellobiose and cellulose) were considered as TS candidates. For PSs, threshold for the ratio of reads in Exo and Hyb was set to 0.5, and only those ESs with the ratio lower than the threshold under <u>each</u> of the conditions where the PS was detected were considered as PS candidates.

(*v*) Producing the final lists of TSs and PSs. Among the candidates derived from the previous step, the final list of TSs was generated by only including those that are of the same orientation to that of their downstream genes. The final list of PSs was generated by removing those that are located at 3' terminal of the operons, which are potential transcriptional terminator.

Supplementary Reference:

1 Blouzard, J. C. *et al.* Modulation of cellulosome composition in Clostridium cellulolyticum: adaptation to the polysaccharide environment revealed by proteomic and carbohydrate-active enzyme analyses. *Proteomics*. **10**, 541-554 (2010).

2 Mitschke, J. *et al.* An experimentally anchored map of transcriptional start sites in the model cyanobacterium Synechocystis sp. PCC6803. *Proc Natl Acad Sci U S A*. **108**, 2124-2129 (2011).

3 Dugar, G. *et al.* High-resolution transcriptome maps reveal strain-specific regulatory features of multiple Campylobacter jejuni isolates. *PLoS Genet*. **9**, e1003495 (2013).

4 Sharma, C. M. *et al.* The primary transcriptome of the major human pathogen Helicobacter pylori. *Nature*. **464**, 250-255 (2010).

5 Jorjani, H. & Zavolan, M. TSSer: an automated method to identify transcription start sites in prokaryotic genomes from differential RNA sequencing data. *Bioinformatics*. **30**, 971-974 (2014).
